# Supplementary material for: Rapid biphasic decay of intact and defective HIV DNA reservoir during acute treated HIV disease
Source: Nat Commun. 2024 Nov 18;15:9966. doi: 10.1038/s41467-024-54116-1 (PMC11574060; doi:10.1038/s41467-024-54116-1)
Supplement: Supplementary file 2 — Description of Additional Supplementary Files [file 41467_2024_54116_MOESM2_ESM.docx]

**Supplementary Data. 1: Code and anonymized clinical data required to replicate analyses and figures.** Intermediate bootstrapping and cross-validation results are included for extra reproducibility and to reduce run time required to reproduce results.
