# Supplementary material for: Rapid biphasic decay of intact and defective HIV DNA reservoir during acute treated HIV disease
Source: Nat Commun. 2024 Nov 18;15:9966. doi: 10.1038/s41467-024-54116-1 (PMC11574060; doi:10.1038/s41467-024-54116-1)
Supplement: Supplementary file 3 — Supplementary Data 1 [file 41467_2024_54116_MOESM3_ESM.zip › Barbehenn 2024 - Supplementary Code and Data/Modeling.html]

Acute HIV Reservoir Decay Modeling


# Acute HIV Reservoir Decay Modeling

#### Alton Barbehenn

#### 2024-10-04

```
library(dplyr)
library(tidyr)
library(readr)
library(lubridate)
library(mgcv)
library(lspline)
library(ggplot2)
library(ggpubr)
library(patchwork)
library(mgcViz)
library(plotly)
library(knitr)
library(parallel)
library(parallelly)

# Set parallel cores for LOOCV
NCORES = unname(parallelly::availableCores()) - 1
```

# Define Models and Create Data sets

```
# chosen parameters
TAU = 5
TAU1 = 0.5
TAU2 = 4

avg_eid_art_days = 59.2836

# original data set
df_full = read_csv("anonymized_merged.csv") %>%
    filter(assay != "PlasmaVL") %>%
    pivot_wider(names_from = assay, values_from = concentration) %>%
    # center eid_art_days using total cohort (df does not necessarily have mean zero eid_art_days after filters are applied)
    mutate(eid_art_days = eid_art_days - avg_eid_art_days) %>%
    mutate(PID = as.factor(PID)) %>% # VERY IMPORTANT because PID may be an integer by default
    rename(days_art_num = days_on_art) %>%
    mutate(eid_art_weeks = eid_art_days / 7) %>%
    mutate(weeks_art_num = days_art_num / 7) %>%
    bind_cols(lspline(.$weeks_art_num, knots = TAU, marginal = FALSE)) %>% 
    rename(phase1 = "1", phase2 = "2") %>%
    filter(`CD4+ T Cell Viability` >= 0.70)


dfpvl_full = read_csv("anonymized_merged.csv") %>%
    filter(assay == "PlasmaVL") %>%
    pivot_wider(names_from = assay, values_from = concentration) %>%
    # center eid_art_days using total cohort (df does not necessarily have mean zero eid_art_days after filters are applied)
    mutate(eid_art_days = eid_art_days - avg_eid_art_days) %>%
    mutate(PID = as.factor(PID)) %>% # VERY IMPORTANT because PID may be an integer by default
    rename(days_art_num = days_on_art) %>%
    filter(days_art_num >= -2) %>%
    mutate(eid_art_weeks = eid_art_days / 7) %>%
    mutate(weeks_art_num = days_art_num / 7) %>%
    bind_cols(lspline(.$weeks_art_num, knots = c(TAU1, TAU2), marginal = FALSE)) %>%
    rename(phase1 = "1", phase2 = "2", phase3 = "3") %>%
    # remove unneeded pre-ART plasma viral loads
    filter(PID != "0727f593-30d5-4a26-95d9-7a854cb2b08d" | days_art_num != -1) %>%
    filter(PID != "07fad2aa-bfba-4075-bf62-97fadda760ae" | days_art_num != -2) %>%
    filter(PID != "9e5d1f5a-8872-40d4-810c-4eba914b185f" | days_art_num != -1)


# main data sets
df = df_full %>%
    filter(days_art_num <= 183)

dfpvl = dfpvl_full %>%
    filter(days_art_num <= 183)
```

```
dfpvl_full %>%
    bind_rows(tribble(~PID, ~weeks_art_num, ~PlasmaVL,
                      "1c7a9276-9420-4195-a974-2f2a9d4530b7", 1000, 0,
                      "1a331b87-3f5d-4ce3-a86b-1673d8a69e58", 0.5, 0)) %>%
    filter(PlasmaVL <= 40) %>%
    slice_min(weeks_art_num, n = 1, by = PID, with_ties = FALSE) %>% 
    summarise(median(weeks_art_num))
```

```
## # A tibble: 1 × 1
##   `median(weeks_art_num)`
##                     <dbl>
## 1                    4.14
```

```
## df_full WITHOUT the filter step for ipda quality
read_csv("anonymized_merged.csv") %>%
    summarise(delta = max(days_on_art, na.rm = TRUE)/365, .by = PID) %>% 
    pull(delta) %>% 
    summary() %>%
    round(2)
```

```
##    Min. 1st Qu.  Median    Mean 3rd Qu.    Max. 
##    0.01    0.48    0.71    0.64    0.84    1.00
```

```
# Biphasic models
## no covariates (unadjusted)
model_formula_unadjusted = paste0(
    "log2(viral_reservoir) ~ ",
    "phase1 + ",
    "phase2 + ",
    "s(PID, bs = 're')"
)

# adjusted for log10firstvl and initial CD4 count
model_formula = paste0(
    "log2(viral_reservoir) ~ ",
    "phase1 + ",
    "phase2 + ",
    "phase1 : eid_art_weeks + ",
    "phase2 : eid_art_weeks + ",
    "s(log10firstvl, bs = 'cr', k = 20) + ",
    "s(first_cd4, bs = 'cr', k = 20) + ",
    "s(PID, bs = 're')"
)

# when modeling plasma viral load, log10firstvl and a random PID intercept are basically redundant
model_formula_pl = paste0(
    "log2(viral_reservoir) ~ ",
    "phase1 + ",
    "phase2 + ",
    "phase1 : eid_art_weeks + ",
    "phase2 : eid_art_weeks + ",
    "s(first_cd4, bs = 'cr', k = 20) + ",
    "s(PID, bs = 're')"
)
```

```
# Triphasic models
## no covariates (unadjusted)
model_formula3_unadjusted = paste0(
    "log2(viral_reservoir) ~ ",
    "phase1 + ",
    "phase2 + ",
    "phase3 + ",
    "s(PID, bs = 're')"
)

# adjusted for log10firstvl and initial CD4 count
model_formula3 = paste0(
    "log2(viral_reservoir) ~ ",
    "phase1 + ",
    "phase2 + ",
    "phase3 + ",
    "phase1 : eid_art_weeks + ",
    "phase2 : eid_art_weeks + ",
    "phase3 : eid_art_weeks + ",
    "s(log10firstvl, bs = 'cr', k = 20) + ",
    "s(first_cd4, bs = 'cr', k = 20) + ",
    "s(PID, bs = 're')"
)

# when modeling plasma viral load, log10firstvl and a random PID intercept are basically redundant
model_formula3_pl = paste0(
    "log2(viral_reservoir) ~ ",
    "phase1 + ",
    "phase2 + ",
    "phase3 + ",
    "phase1 : eid_art_weeks + ",
    "phase2 : eid_art_weeks + ",
    "phase3 : eid_art_weeks + ",
    "s(first_cd4, bs = 'cr', k = 20) + ",
    "s(PID, bs = 're')"
)
```

## Helper Function

```
# NOTE: half-life is in units 1/time model time scale, no transformations are included.
halflife = function(mod, phase) {
    # variable names
    phb  = paste0("phase", phase)
    phd = paste0(phb, ":eid_art_weeks")
    
    # estimated means and variances from model
    b = summary(mod)$p.coeff[[phb]]
    d = summary(mod)$p.coeff[[phd]]
    Sigma = vcov.gam(mod)[c(phb, phd), c(phb, phd)]
    
    # Jacobian matrix
    J = matrix(c(
        1/b^2,     0,
        -2*d/b^3,  1/b^2
        ), nrow = 2, ncol = 2, byrow = TRUE,
        dimnames = list(c(phb, phd), c(phb, phd)))
    
    # compute halflife and halflife adjustment and std.errors
    tribble(
               ~transform, ~term, ~estimate,                           ~std.error,
         "asymp.halflife",   phb,      -1/b, sqrt(J %*% Sigma %*% t(J))[phb, phb],
         "asymp.halflife",   phd,     d/b^2, sqrt(J %*% Sigma %*% t(J))[phd, phd]
    )
}
```

# Model Phase Tuning/Estimation (0-24 Weeks)

## Monophasic Models

```
# only one model, so there's no need for fancy functions
gam_tuning_loocv_1phase = function() {
    predictionMAE_Intact = list()
    predictionMSE_Intact = list()
    predictionMAE_Defective = list()
    predictionMSE_Defective = list()
    predictionMAE_PlasmaVL = list()
    predictionMSE_PlasmaVL = list()
    
    # Leave-one-out cross-validation by PID
    for (pid in levels(df$PID)) {
        ## for intact DNA analysis
        mod_int_mono = gam(
            formula = as.formula(paste0(
                "log2(Intact) ~ ",
                "weeks_art_num + ",
                "weeks_art_num : eid_art_weeks + ",
                "s(log10firstvl, bs = 'cr', k = 20) + ",
                "s(first_cd4, bs = 'cr', k = 20) + ",
                "s(PID, bs = 're')")),
            data = df %>% filter(PID != pid),
            method = "REML"
        )
        
        ## for defective DNA analysis
        mod_def_mono = gam(
            formula = as.formula(paste0(
                "log2(Defective) ~ ",
                "weeks_art_num + ",
                "weeks_art_num : eid_art_weeks + ",
                "s(log10firstvl, bs = 'cr', k = 20) + ",
                "s(first_cd4, bs = 'cr', k = 20) + ",
                "s(PID, bs = 're')")),
            data = df %>% filter(PID != pid),
            method = "REML"
        )

        ## for plasma viral load analysis
        mod_pl_mono = gam(
            formula = as.formula(paste0(
                "log2(PlasmaVL) ~ ",
                "weeks_art_num + ",
                "weeks_art_num : eid_art_weeks + ",
                "s(first_cd4, bs = 'cr', k = 20) + ",
                "s(PID, bs = 're')")),
            data = dfpvl %>% filter(PID != pid),
            method = "REML"
        )
        
        predictionMAE_Intact[[pid]] = mean(abs(predict(mod_int_mono, newdata = df %>% filter(PID == pid)) - log2(df %>% filter(PID == pid) %>% pull(Intact))), na.rm = TRUE)
        predictionMSE_Intact[[pid]] = mean((predict(mod_int_mono, newdata = df %>% filter(PID == pid)) - log2(df %>% filter(PID == pid) %>% pull(Intact)))^2, na.rm = TRUE)
        predictionMAE_Defective[[pid]] = mean(abs(predict(mod_def_mono, newdata = df %>% filter(PID == pid)) - log2(df %>% filter(PID == pid) %>% pull(Defective))), na.rm = TRUE)
        predictionMSE_Defective[[pid]] = mean((predict(mod_def_mono, newdata = df %>% filter(PID == pid)) - log2(df %>% filter(PID == pid) %>% pull(Defective)))^2, na.rm = TRUE)
        predictionMAE_PlasmaVL[[pid]] = mean(abs(predict(mod_pl_mono, newdata = dfpvl %>% filter(PID == pid)) - log2(dfpvl %>% filter(PID == pid) %>% pull(PlasmaVL))), na.rm = TRUE)
        predictionMSE_PlasmaVL[[pid]] = mean((predict(mod_pl_mono, newdata = dfpvl %>% filter(PID == pid)) - log2(dfpvl %>% filter(PID == pid) %>% pull(PlasmaVL)))^2, na.rm = TRUE)
    }
    
    # Fit model with all data
    mod_int_mono = gam(
        formula = as.formula(paste0(
            "log2(Intact) ~ ",
            "weeks_art_num + ",
            "weeks_art_num : eid_art_weeks + ",
            "s(log10firstvl, bs = 'cr', k = 20) + ",
            "s(first_cd4, bs = 'cr', k = 20) + ",
            "s(PID, bs = 're')")),
        family = gaussian(),
        data = df,
        method = "REML"
    )
    
    mod_def_mono = gam(
        formula = as.formula(paste0(
            "log2(Defective) ~ ",
            "weeks_art_num + ",
            "weeks_art_num : eid_art_weeks + ",
            "s(log10firstvl, bs = 'cr', k = 20) + ",
            "s(first_cd4, bs = 'cr', k = 20) + ",
            "s(PID, bs = 're')")),
        family = gaussian(),
        data = df,
        method = "REML"
    )

    mod_pl_mono = gam(
        formula = as.formula(paste0(
            "log2(PlasmaVL) ~ ",
            "weeks_art_num + ",
            "weeks_art_num : eid_art_weeks + ",
            "s(first_cd4, bs = 'cr', k = 20) + ",
            "s(PID, bs = 're')")),
        family = gaussian(),
        data = dfpvl,
        method = "REML"
    )
    
    cbind(predictionMAE_Intact = mean(as.numeric(predictionMAE_Intact), na.rm = TRUE),
          predictionMAEsd_Intact = sd(as.numeric(predictionMAE_Intact), na.rm = TRUE),
          predictionMSE_Intact = mean(as.numeric(predictionMSE_Intact), na.rm = TRUE),
          predictionMSEsd_Intact = sd(as.numeric(predictionMSE_Intact), na.rm = TRUE),
          AIC_Intact = AIC(mod_int_mono),
          adjR2_Intact = summary(mod_int_mono)$r.sq,
          devExp_Intact = summary(mod_int_mono)$dev.expl,
          predictionMAE_Defective = mean(as.numeric(predictionMAE_Defective), na.rm = TRUE),
          predictionMAEsd_Defective = sd(as.numeric(predictionMAE_Defective), na.rm = TRUE),
          predictionMSE_Defective = mean(as.numeric(predictionMSE_Defective), na.rm = TRUE),
          predictionMSEsd_Defective = sd(as.numeric(predictionMSE_Defective), na.rm = TRUE),
          AIC_Defective = AIC(mod_def_mono),
          adjR2_Defective = summary(mod_def_mono)$r.sq,
          devExp_Defective = summary(mod_def_mono)$dev.expl,
          predictionMAE_PlasmaVL = mean(as.numeric(predictionMAE_PlasmaVL), na.rm = TRUE),
          predictionMAEsd_PlasmaVL = sd(as.numeric(predictionMAE_PlasmaVL), na.rm = TRUE),
          predictionMSE_PlasmaVL = mean(as.numeric(predictionMSE_PlasmaVL), na.rm = TRUE),
          predictionMSEsd_PlasmaVL = sd(as.numeric(predictionMSE_PlasmaVL), na.rm = TRUE),
          AIC_PlasmaVL = AIC(mod_pl_mono),
          adjR2_PlasmaVL = summary(mod_pl_mono)$r.sq,
          devExp_PlasmaVL = summary(mod_pl_mono)$dev.expl) %>%
        as_tibble() %>%
        pivot_longer(cols = everything()) %>%
        separate(name, sep = "_", into = c("metric", "assay"))
}

gam_tuning_loocv_1phase() %>%
    write_csv("loocv_tuning_monophasic.csv")
```

## Biphasic Models

```
#' Leave-one-out model cross-validation and AIC calculations
#' @param tau a non-negative knot/inflection point
#' @param dat_ipda the model data frame for IPDA models
#' @param dat_pl the model data frame for plasma viral load models
#' @param ncores the parallelization used for the LOOCV model evaluation
gam_tuning_loocv_2phase = function (tau, dat_ipda = df, dat_pl = dfpvl, ncores = 1L) {
    
    PIDS = levels(dat_pl$PID)
    df_tuning = dat_ipda %>%
        select(-starts_with("phase")) %>%
        bind_cols(lspline(.$weeks_art_num, knots = tau, marginal = FALSE)) %>% 
        rename(phase1 = "1", phase2 = "2")
    dfpvl_tuning = dat_pl %>%
        select(-starts_with("phase")) %>%
        bind_cols(lspline(.$weeks_art_num, knots = tau, marginal = FALSE)) %>% 
        rename(phase1 = "1", phase2 = "2")

    loocv_res = parallel::mclapply(seq_along(PIDS), FUN = function(i) {
        # Leave-one-out cross-validation split
        training_hiv = df_tuning %>% filter(PID != PIDS[i])
        testing_hiv  = df_tuning %>% filter(PID == PIDS[i])
        training_hiv_pl = dfpvl_tuning %>% filter(PID != PIDS[i])
        testing_hiv_pl  = dfpvl_tuning %>% filter(PID == PIDS[i])
        
        ## for intact DNA analysis
        fit_Intact = gam(
            as.formula(sub("viral_reservoir", "Intact", model_formula)),
            data = training_hiv,
            method = "REML"
        )
        
        ## for defective DNA analysis
        fit_Defective = gam(
            as.formula(sub("viral_reservoir", "Defective", model_formula)),
            data = training_hiv,
            method = "REML"
        )
        
        ## for defective DNA analysis
        fit_PlasmaVL = gam(
            as.formula(sub("viral_reservoir", "PlasmaVL", model_formula_pl)),
            data = training_hiv_pl,
            method = "REML"
        )
        
        list(
            "predictionMAE_Intact" = mean(abs(predict(fit_Intact, newdata = testing_hiv) - log2(testing_hiv$Intact)), na.rm = TRUE),
            "predictionMSE_Intact" = mean((predict(fit_Intact, newdata = testing_hiv) - log2(testing_hiv$Intact))^2, na.rm = TRUE),
            "predictionMAE_Defective" = mean(abs(predict(fit_Defective, newdata = testing_hiv) - log2(testing_hiv$Defective)), na.rm = TRUE),
            "predictionMSE_Defective" = mean((predict(fit_Defective, newdata = testing_hiv) - log2(testing_hiv$Defective))^2, na.rm = TRUE),
            "predictionMAE_PlasmaVL" = mean(abs(predict(fit_PlasmaVL, newdata = testing_hiv_pl) - log2(testing_hiv_pl$PlasmaVL)), na.rm = TRUE),
            "predictionMSE_PlasmaVL" = mean((predict(fit_PlasmaVL, newdata = testing_hiv_pl) - log2(testing_hiv_pl$PlasmaVL))^2, na.rm = TRUE)
        )
    }, mc.cores = ncores)

    # collect results
    keys = names(loocv_res[[1]])
    loocv_res = do.call(mapply, c(FUN=c, lapply(loocv_res, `[`, keys)))

    # fit full models for AIC
    fit_Intact = gam(
        as.formula(sub("viral_reservoir", "Intact", model_formula)),
        data = df_tuning,
        method = "REML"
    )
    
    fit_Defective = gam(
        as.formula(sub("viral_reservoir", "Defective", model_formula)),
        data = df_tuning,
        method = "REML"
    )
    
    fit_PlasmaVL = gam(
        as.formula(sub("viral_reservoir", "PlasmaVL", model_formula_pl)),
        data = dfpvl_tuning,
        method = "REML"
    )

    # summarize results
    tibble(predictionMAE_Intact = mean(loocv_res[,"predictionMAE_Intact"], na.rm = TRUE),
           predictionMAEsd_Intact = sd(loocv_res[,"predictionMAE_Intact"], na.rm = TRUE),
           predictionMSE_Intact = mean(loocv_res[,"predictionMSE_Intact"], na.rm = TRUE),
           predictionMSEsd_Intact = sd(loocv_res[,"predictionMSE_Intact"], na.rm = TRUE),
           AIC_Intact = AIC(fit_Intact),
           adjR2_Intact = summary(fit_Intact)$r.sq,
           devExp_Intact = summary(fit_Intact)$dev.expl,
           predictionMAE_Defective = mean(loocv_res[,"predictionMAE_Defective"], na.rm = TRUE),
           predictionMAEsd_Defective = sd(loocv_res[,"predictionMAE_Defective"], na.rm = TRUE),
           predictionMSE_Defective = mean(loocv_res[,"predictionMSE_Defective"], na.rm = TRUE),
           predictionMSEsd_Defective = sd(loocv_res[,"predictionMSE_Defective"], na.rm = TRUE),
           AIC_Defective = AIC(fit_Defective),
           adjR2_Defective = summary(fit_Defective)$r.sq,
           devExp_Defective = summary(fit_Defective)$dev.expl,
           predictionMAE_PlasmaVL = mean(loocv_res[,"predictionMAE_PlasmaVL"], na.rm = TRUE),
           predictionMAEsd_PlasmaVL = sd(loocv_res[,"predictionMAE_PlasmaVL"], na.rm = TRUE),
           predictionMSE_PlasmaVL = mean(loocv_res[,"predictionMSE_PlasmaVL"], na.rm = TRUE),
           predictionMSEsd_PlasmaVL = sd(loocv_res[,"predictionMSE_PlasmaVL"], na.rm = TRUE),
           AIC_PlasmaVL = AIC(fit_PlasmaVL),
           adjR2_PlasmaVL = summary(fit_PlasmaVL)$r.sq,
           devExp_PlasmaVL = summary(fit_PlasmaVL)$dev.expl) %>%
        pivot_longer(cols = everything()) %>%
        separate(name, sep = "_", into = c("metric", "assay"))
}

tibble(tau = (0:54)/2) %>%
    reframe(gam_tuning_loocv_2phase(tau, dat_ipda = df, dat_pl = dfpvl, ncores = NCORES), .by = tau) %>%
    write_csv("loocv_tuning_biphasic.csv")
```

```
read_csv("loocv_tuning_biphasic.csv") %>%
    filter(tau < 25) %>%
    filter(metric == "predictionMAE") %>%
    slice_min(value, by = assay) %>%
    kable()
```

| tau | metric | assay | value |
| --- | --- | --- | --- |
| 5.0 | predictionMAE | Intact | 2.144467 |
| 3.5 | predictionMAE | Defective | 1.710742 |
| 3.5 | predictionMAE | PlasmaVL | 1.657378 |

```
read_csv("loocv_tuning_biphasic.csv") %>%
    filter(tau <= 25) %>%
    filter(metric %in% c("predictionMAE", "predictionMSE")) %>%
    mutate(metric = factor(metric, levels = c("predictionMAE", "predictionMSE"), labels = c("Prediction MAE", "Prediction MSE"))) %>%
    filter(assay %in% c("Intact", "Defective")) %>%
    mutate(assay = factor(assay, levels = c("Intact", "Defective", "PlasmaVL"), labels = c("HIV Intact DNA", "HIV Defective DNA", "HIV Plasma RNA"))) %>%
    mutate(group = factor(paste(assay, metric, sep = ": "))) %>%
    mutate(group = factor(group, levels = levels(group)[c(3,1,4,2)])) %>%
    mutate(ismin = value == min(value), .by = c(metric, assay)) %>%
    ggplot(mapping = aes(x = tau, y = value, color = ismin)) +
    facet_wrap(vars(group), scales = "free_y") +
    geom_point() + 
    geom_vline(xintercept = TAU, linetype = "dashed") +
    scale_x_continuous(name = expression(paste("Inflection Point" ~ tau ~ "(Weeks on ART)")), breaks = (0:6)*4) +
    scale_y_continuous(name = "Loss Metric") +
    scale_color_manual(name = "Loss Metric Minima", values = c("TRUE" = "red", "FALSE" = "black")) +
    theme_pubr(base_size = 6, base_family = "Helvetica") +
    theme(legend.box.spacing = unit(0, "mm"))
```

```
ggsave("figs/main/loocv_ipda_model_tuning_biphasic.pdf", width = 88, height = 70, units = "mm")
```

```
# proposed model
mod_int = gam(
    formula = as.formula(sub("viral_reservoir", "Intact", model_formula)),
    family = gaussian(),
    data = df,
    method = "REML"
)

mod_def = gam(
    formula = as.formula(sub("viral_reservoir", "Defective", model_formula)),
    family = gaussian(),
    data = df,
    method = "REML"
)

mod_def3 = gam(
    formula = as.formula(sub("viral_reservoir", "Defective3", model_formula)),
    family = gaussian(),
    data = df,
    method = "REML"
)

mod_def5 = gam(
    formula = as.formula(sub("viral_reservoir", "Defective5", model_formula)),
    family = gaussian(),
    data = df,
    method = "REML"
)
```

```
n_i = summary(mod_int)$residual.df; e_i = -0.353617; s_i = 0.027982/sqrt(n_i)
n_d = summary(mod_def)$residual.df; e_d = -0.735168; s_d = 0.051570/sqrt(n_d)
sdx = sqrt(s_i*s_i + s_d*s_d)
2+pt(abs(e_i - e_d) / sdx, 
   df = (s_i^2 + s_d^2)^2 / (s_i^4/(n_i-1) + s_d^4/(n_d-1)),
   lower.tail = FALSE, log.p = TRUE)
```

```
## [1] -801.8166
```

```
ggarrange(
    df %>%
        summarise(eid_art_weeks = first(eid_art_weeks),
                  log10firstvl = first(log10firstvl),
                  first_cd4 = first(first_cd4),
                  .by = PID) %>%
        select(-PID) %>%
        summarise(across(everything(), mean)) %>%
        expand_grid(weeks_art_num = (0:183) / 7) %>%
        mutate(PID = as.factor("a")) %>% # unknown/zero random effect
        bind_cols(lspline(.$weeks_art_num, knots = TAU, marginal = FALSE)) %>% 
        rename(phase1 = "1", phase2 = "2") %>% 
        mutate(Intact = 2^predict(mod_int, newdata = .)) %>%
        mutate(Defective = 2^predict(mod_def, newdata = .)) %>%
        pivot_longer(cols = c(Intact, Defective)) %>%
        mutate(name = factor(name, levels = c("Intact", "Defective"), labels = c("HIV Intact DNA", "HIV Defective DNA"))) %>%
        ggplot(aes(x = weeks_art_num, y = value)) +
        facet_wrap(~name, scales = "free_y") +
        geom_line(mapping = aes(x = weeks_art_num, y = value, group = PID), 
                  data = df %>% 
                      pivot_longer(c(Intact, Defective)) %>% 
                      mutate(name = factor(name, levels = c("Intact", "Defective"), labels = c("HIV Intact DNA", "HIV Defective DNA"))),
                  color = "lightgrey", alpha = 0.5) +
        geom_line() +
        scale_x_continuous(name = "Sampling Timepoints (Weeks on ART)", breaks = c(2,(0:6)*4)) +
        scale_y_log10(name = expression(atop("HIV DNA", paste("(copies/", 10^6, " CD4+ T cells)")))) +
        theme_pubr(base_size = 6, base_family = "Helvetica"),
    df %>%
        summarise(eid_art_weeks = first(eid_art_weeks),
                  log10firstvl = first(log10firstvl),
                  first_cd4 = first(first_cd4),
                  .by = PID) %>%
        select(-PID) %>%
        summarise(across(everything(), mean)) %>%
        expand_grid(weeks_art_num = (0:183) / 7) %>%
        mutate(PID = as.factor("a")) %>% # unknown/zero random effect
        bind_cols(lspline(.$weeks_art_num, knots = TAU, marginal = FALSE)) %>% 
        rename(phase1 = "1", phase2 = "2") %>% 
        mutate(Defective3 = 2^predict(mod_def3, newdata = .)) %>%
        mutate(Defective5 = 2^predict(mod_def5, newdata = .)) %>%
        pivot_longer(cols = c(Defective3, Defective5)) %>%
        mutate(name = factor(name, levels = c("Defective3", "Defective5"), labels = c("HIV Defective 3' DNA", "HIV Defective 5' DNA"))) %>%
        ggplot(aes(x = weeks_art_num, y = value)) +
        facet_wrap(~name, scales = "free_y") +
        geom_line(mapping = aes(x = weeks_art_num, y = value, group = PID), 
                  data = df %>% 
                      pivot_longer(c(Defective3, Defective5)) %>% 
                      mutate(name = factor(name, levels = c("Defective3", "Defective5"), labels = c("HIV Defective 3' DNA", "HIV Defective 5' DNA"))), 
                  color = "lightgrey", alpha = 0.5) +
        geom_line() +
        scale_x_continuous(name = "Sampling Timepoints (Weeks on ART)", breaks = c(2,(0:6)*4)) +
        scale_y_log10(name = expression(atop("HIV DNA", paste("(copies/", 10^6, " CD4+ T cells)")))) +
        theme_pubr(base_size = 6, base_family = "Helvetica"),
    nrow = 2,
    labels = c("a.", "b."),
    hjust = -0.1
)
```

```
ggsave("figs/main/predicted_average_patient_model_all.pdf", width = 88, height = 66, units = "mm")
```

```
ggarrange(
    gridPrint(
        plot(sm(getViz(mod_int), 1)) + 
            l_points(shape = 19, size = 1, alpha = 0.5) + 
            l_fitLine(colour = "red") + 
            l_ciLine(color = "blue") +
            l_rug(mapping = aes(x=x, y=y), alpha = 1) + 
            labs(x = expression(Log[10]*" Pre-ART Viral Load (copies/mL)"),
                 y = expression(atop("Additive Effect to the",Log[2]*" HIV Intact DNA"))) +
            theme_pubr(base_size = 10, base_family = "Helvetica"),
        plot(sm(getViz(mod_int), 2)) +
            l_points(shape = 19, size = 1, alpha = 0.5) + 
            l_fitLine(colour = "red") +
            l_ciLine(color = "blue") +
            l_rug(mapping = aes(x= x, y=y), alpha = 1) +
            labs(x = expression("Initial CD4+ T Cell Count (Cells/"*mm^3*")"),
                 y = expression(atop("Additive Effect to the",Log[2]*" HIV Intact DNA"))) +
            theme_pubr(base_size = 10, base_family = "Helvetica"),
        nrow = 1
    ),
    gridPrint(
        plot(sm(getViz(mod_def), 1)) + 
            l_points(shape = 19, size = 1, alpha = 0.5) + 
            l_fitLine(colour = "red") + 
            l_ciLine(color = "blue") +
            l_rug(mapping = aes(x=x, y=y), alpha = 1) + 
            labs(x = expression(Log[10]*" Pre-ART Viral Load (copies/mL)"),
                 y = expression(atop("Additive Effect to the",Log[2]*" HIV Defective DNA"))) +
            theme_pubr(base_size = 10, base_family = "Helvetica"),
        plot(sm(getViz(mod_def), 2)) +
            l_points(shape = 19, size = 1, alpha = 0.5) + 
            l_fitLine(colour = "red") +
            l_ciLine(color = "blue") +
            l_rug(mapping = aes(x=x, y=y), alpha = 1) +
            labs(x = expression("Initial CD4+ T Cell Count (Cells/"*mm^3*")"),
                 y = expression(atop("Additive Effect to the",Log[2]*" HIV Defective DNA"))) +
            theme_pubr(base_size = 10, base_family = "Helvetica"),
        nrow = 1
    ),
    nrow = 2,
    labels = c("a.", "b."),
    hjust = -0.2,
    vjust = 1.5
)
```

```
ggsave("figs/supp/spline_terms_ipda.pdf", width = 6.5, height = 6, units = "in")
```

```
bind_rows(
    tibble(Assay = "Intact", log2obs = mod_int$model$`log2(Intact)`, log2pred = fitted(mod_int)),
    # tibble(Assay = "Defective 3'", log2obs = mod_def3$model$`log2(Defective3)`, log2pred = fitted(mod_def3)),
    # tibble(Assay = "Defective 5'", log2obs = mod_def5$model$`log2(Defective5)`, log2pred = fitted(mod_def5)),
    tibble(Assay = "Defective", log2obs = mod_def$model$`log2(Defective)`, log2pred = fitted(mod_def)),
    # tibble(Assay = "Plasma VL", log2obs = mod_pl$model$`log2(PlasmaVL)`, log2pred = fitted(mod_pl))
    ) %>%
    mutate(Assay = factor(Assay, levels = c("Intact", "Defective", "Defective 3'", "Defective 5'", "Plasma VL"), labels = c("HIV Intact DNA", "HIV Defective DNA", "HIV Defective 3' DNA", "HIV Defective 5' DNA", "HIV Plasma RNA"))) %>%
    ggplot(aes(x = 2^(log2obs), y = 2^(log2pred))) + 
    facet_wrap(~Assay) +
    geom_point() + 
    geom_abline(slope = 1, intercept = 0, color = "red", linetype = "dashed") +
    xlab(expression(atop("Observed HIV DNA", "(copies /" * 10^6 * " CD4+ T Cells)"))) + 
    ylab(expression(atop("Predicted HIV DNA", "(copies /" * 10^6 * " CD4+ T Cells)"))) + 
    scale_x_log10() + scale_y_log10() +
    theme_pubr(base_size = 11, base_family = "Helvetica")
```

```
ggsave("figs/supp/diagnostic_pred_vs_fitted_ipda.pdf", width = 6.5, height = 3, units = "in")
```

```
# create random patients, then predict their reservoir decays
set.seed(1)
df_sim = df %>%
    select(PID, eid_art_days, first_cd4, log10firstvl) %>%
    distinct() %>%
    sample_n(size = 300, replace = TRUE) %>% # random rows
    mutate(across(everything(), sample)) # shuffle each column (break existing patient relationship)

ggarrange(
    df_sim %>% 
        arrange(eid_art_days) %>% # sort rows/patients by variable of interest
        mutate(tertile = rep(c("First", "Second", "Third"), each = n()/3)) %>% # identify tertiles
        mutate(LB = signif(min(eid_art_days)/7 + avg_eid_art_days/7, digits = 2), UB = signif(max(eid_art_days)/7 + avg_eid_art_days/7, digits = 2), .by = tertile) %>% # make pretty labels for tertiles
        mutate(tertile = factor(tertile, 
                                levels = c("First", "Second", "Third"),
                                labels = unique(paste0(tertile, " Tertile\n(", LB, "-", UB, "]")))) %>%
        expand_grid(weeks_art_num = (0:183) / 7) %>%
        mutate(eid_art_weeks = eid_art_days / 7) %>%
        bind_cols(lspline(.$weeks_art_num, knots = TAU, marginal = FALSE)) %>% 
        rename(phase1 = "1", phase2 = "2") %>% 
        mutate(Intact = as.numeric(predict(mod_int, .)),
               Defective = as.numeric(predict(mod_def, .))) %>% # predict each patient's decay pattern
        mutate(Intact = 2^Intact, Defective = 2^Defective) %>%
        pivot_longer(cols = c(Intact, Defective), names_to = "assay", values_to = "count") %>%
        mutate(assay = factor(assay, levels = c("Intact", "Defective"), labels = c("HIV Intact DNA", "HIV Defective DNA"))) %>%
        ggplot(aes(x = weeks_art_num, y = count)) +
        facet_wrap(~assay, scales = "free_y") +
        geom_smooth(aes(color = tertile)) +
        scale_x_continuous(name = "Sampling Timepoints (Weeks on ART)", breaks = c(2, (0:6)*4)) +
        scale_y_log10(name = expression(atop("HIV DNA", paste("(copies/", 10^6, " CD4+ T cells)")))) +
        scale_color_discrete(name = "Estimated Time Between\nHIV Infection and ART\nInitiation (Weeks)") +
        theme_pubr(base_size = 5, base_family = "Helvetica"),
    df_sim %>% 
        arrange(first_cd4) %>% # sort rows/patients by variable of interest
        mutate(tertile = rep(c("First", "Second", "Third"), each = n()/3)) %>% # identify tertiles
        mutate(LB = signif(min(first_cd4), digits = 2), UB = signif(max(first_cd4), digits = 2), .by = tertile) %>% # make pretty labels for tertiles
        mutate(tertile = factor(tertile, 
                                levels = c("First", "Second", "Third"),
                                labels = unique(paste0(tertile, " Tertile\n(", LB, "-", UB, "]")))) %>%
        expand_grid(weeks_art_num = (0:183) / 7) %>%
        mutate(eid_art_weeks = eid_art_days / 7) %>%
        bind_cols(lspline(.$weeks_art_num, knots = TAU, marginal = FALSE)) %>% 
        rename(phase1 = "1", phase2 = "2") %>% 
        mutate(Intact = as.numeric(predict(mod_int, .)),
               Defective = as.numeric(predict(mod_def, .))) %>% # predict each patient's decay pattern
        mutate(Intact = 2^Intact, Defective = 2^Defective) %>%
        pivot_longer(cols = c(Intact, Defective), names_to = "assay", values_to = "count") %>%
        mutate(assay = factor(assay, levels = c("Intact", "Defective"), labels = c("HIV Intact DNA", "HIV Defective DNA"))) %>%
        ggplot(aes(x = weeks_art_num, y = count)) +
        facet_wrap(~assay, scales = "free_y") +
        geom_smooth(aes(color = tertile)) +
        scale_x_continuous(name = "Sampling Timepoints (Weeks on ART)", breaks = c(2, (0:6)*4)) +
        scale_y_log10(name = expression(atop("HIV DNA", paste("(copies/", 10^6, " CD4+ T cells)")))) +
        scale_color_discrete(name = expression("Initial CD4 Count (Cells/"*mm^3*")")) +
        theme_pubr(base_size = 5, base_family = "Helvetica"),
    df_sim %>% 
        arrange(log10firstvl) %>% # sort rows/patients by variable of interest
        mutate(tertile = rep(c("First", "Second", "Third"), each = n()/3)) %>% # identify tertiles
        mutate(LB = signif(min(log10firstvl), digits = 2), UB = signif(max(log10firstvl), digits = 2), .by = tertile) %>% # make pretty labels for tertiles
        mutate(tertile = factor(tertile, 
                                levels = c("First", "Second", "Third"),
                                labels = unique(paste0(tertile, " Tertile\n(", LB, "-", UB, "]")))) %>%
        expand_grid(weeks_art_num = (0:183) / 7) %>%
        mutate(eid_art_weeks = eid_art_days / 7) %>%
        bind_cols(lspline(.$weeks_art_num, knots = TAU, marginal = FALSE)) %>% 
        rename(phase1 = "1", phase2 = "2") %>% 
        mutate(Intact = as.numeric(predict(mod_int, .)),
               Defective = as.numeric(predict(mod_def, .))) %>% # predict each patient's decay pattern
        mutate(Intact = 2^Intact, Defective = 2^Defective) %>%
        pivot_longer(cols = c(Intact, Defective), names_to = "assay", values_to = "count") %>%
        mutate(assay = factor(assay, levels = c("Intact", "Defective"), labels = c("HIV Intact DNA", "Defective HIV DNA"))) %>%
        ggplot(aes(x = weeks_art_num, y = count)) +
        facet_wrap(~assay, scales = "free_y") +
        geom_smooth(aes(color = tertile)) +
        scale_x_continuous(name = "Sampling Timepoints (Weeks on ART)", breaks = c(2, (0:6)*4)) +
        scale_y_log10(name = expression(atop("HIV DNA", paste("(copies/", 10^6, " CD4+ T cells)")))) +
        scale_color_discrete(name = expression(atop("Pre-ART Viral Load", "(" * Log[10] ~ "copies/mL)"))) +
        theme_pubr(base_size = 5, base_family = "Helvetica"),
    nrow = 3,
    labels = c("a.", "b.", "c.")
)
```

```
ggsave("figs/main/simulated_predicted_ipda_decays.pdf", width = 88, height = 150, units = "mm")
```

```
tribble(
    ~Outcome,       ~Phase,                       ~N_participants,                    ~Estimate,                            ~SE,                              ~p_value,
    "Intact",       paste0("0-", TAU, " Weeks"),  length(unique(mod_int$model$PID)),  summary(mod_int)$p.coef[["phase1"]],  summary(mod_int)$se[["phase1"]],  summary(mod_int)$p.pv[["phase1"]],
    "Defective",    paste0("0-", TAU, " Weeks"),  length(unique(mod_def$model$PID)),  summary(mod_def)$p.coef[["phase1"]],  summary(mod_def)$se[["phase1"]],  summary(mod_def)$p.pv[["phase1"]],
    "Defective 3'", paste0("0-", TAU, " Weeks"),  length(unique(mod_def3$model$PID)), summary(mod_def3)$p.coef[["phase1"]], summary(mod_def3)$se[["phase1"]], summary(mod_def3)$p.pv[["phase1"]],
    "Defective 5'", paste0("0-", TAU, " Weeks"),  length(unique(mod_def5$model$PID)), summary(mod_def5)$p.coef[["phase1"]], summary(mod_def5)$se[["phase1"]], summary(mod_def5)$p.pv[["phase1"]],
    "Intact",       paste0(TAU, "-24 Weeks"),     length(unique(mod_int$model$PID)),  summary(mod_int)$p.coef[["phase2"]],  summary(mod_int)$se[["phase2"]],  summary(mod_int)$p.pv[["phase2"]], 
    "Defective",    paste0(TAU, "-24 Weeks"),     length(unique(mod_def$model$PID)),  summary(mod_def)$p.coef[["phase2"]],  summary(mod_def)$se[["phase2"]],  summary(mod_def)$p.pv[["phase2"]],
    "Defective 3'", paste0(TAU, "-24 Weeks"),     length(unique(mod_def3$model$PID)), summary(mod_def3)$p.coef[["phase2"]], summary(mod_def3)$se[["phase2"]], summary(mod_def3)$p.pv[["phase2"]],
    "Defective 5'", paste0(TAU, "-24 Weeks"),     length(unique(mod_def5$model$PID)), summary(mod_def5)$p.coef[["phase2"]], summary(mod_def5)$se[["phase2"]], summary(mod_def5)$p.pv[["phase2"]]) %>%
    mutate(PercentDecayRatePerWeek = -100*(2^Estimate - 1)) %>% # negative because it's in terms of decay rather than percent change
    # mutate(halflife_month = -1/Estimate/4) %>%
    # mutate(halflifesd = 1/Estimate^2*SE/4) %>%
    # mutate(halflife_lowCI = halflife_month - 1.96*halflifesd, halflife_highCI = halflife_month + 1.96*halflifesd) %>%
    mutate(halflife_week = -1/Estimate) %>%
    mutate(halflifesd = 1/Estimate^2*SE) %>%
    mutate(halflife_lowCI = halflife_week - 1.96*halflifesd, halflife_highCI = halflife_week + 1.96*halflifesd) %>%
    # mutate(halflife_days = -1/Estimate*7) %>%
    # mutate(halflifesd = 1/Estimate^2*SE*7) %>%
    # mutate(halflife_lowCI = halflife_days - 1.96*halflifesd, halflife_highCI = halflife_days + 1.96*halflifesd) %>%
    mutate(across(where(is.double), ~format(.x, digits = 4))) %>%
    select(-halflifesd) %>% 
    kable()
```

| Outcome | Phase | N\_participants | Estimate | SE | p\_value | PercentDecayRatePerWeek | halflife\_week | halflife\_lowCI | halflife\_highCI |
| --- | --- | --- | --- | --- | --- | --- | --- | --- | --- |
| Intact | 0-5 Weeks | 61 | -0.353617 | 0.027982 | 1.077e-29 | 21.7380 | 2.8279 | 2.3893 | 3.2665 |
| Defective | 0-5 Weeks | 61 | -0.735168 | 0.051570 | 8.254e-36 | 39.9252 | 1.3602 | 1.1732 | 1.5472 |
| Defective 3’ | 0-5 Weeks | 61 | -0.179619 | 0.083671 | 3.257e-02 | 11.7064 | 5.5674 | 0.4842 | 10.6505 |
| Defective 5’ | 0-5 Weeks | 61 | -1.238645 | 0.085613 | 9.917e-37 | 57.6230 | 0.8073 | 0.6980 | 0.9167 |
| Intact | 5-24 Weeks | 61 | -0.064824 | 0.007418 | 1.635e-16 | 4.3938 | 15.4265 | 11.9666 | 18.8864 |
| Defective | 5-24 Weeks | 61 | 0.006558 | 0.013704 | 6.326e-01 | -0.4556 | -152.4931 | -777.1006 | 472.1145 |
| Defective 3’ | 5-24 Weeks | 61 | 0.018784 | 0.022244 | 3.991e-01 | -1.3105 | -53.2366 | -176.7997 | 70.3265 |
| Defective 5’ | 5-24 Weeks | 61 | 0.039987 | 0.022760 | 7.990e-02 | -2.8105 | -25.0079 | -52.9065 | 2.8906 |

## Triphasic Models

```
#' Leave-one-out model cross-validation and AIC calculations
#' @param tau1 a first non-negative knot/inflection point
#' @param tau2 a second non-negative knot/inflection point
#' @param dat_ipda the model data frame for IPDA models
#' @param dat_pl the model data frame for plasma viral load models
#' @param ncores the parallelization used for the LOOCV model evaluation
gam_tuning_loocv_3phase = function (tau1, tau2, dat_ipda = df, dat_pl = dfpvl, ncores = 1L) {
    
    PIDS = levels(dat_pl$PID)
    df_tuning = dat_ipda %>%
        select(-starts_with("phase")) %>%
        bind_cols(lspline(.$weeks_art_num, knots = c(tau1, tau2), marginal = FALSE)) %>% 
        rename(phase1 = "1", phase2 = "2", phase3 = "3")
    dfpvl_tuning = dat_pl %>%
        select(-starts_with("phase")) %>%
        bind_cols(lspline(.$weeks_art_num, knots = c(tau1, tau2), marginal = FALSE)) %>% 
        rename(phase1 = "1", phase2 = "2", phase3 = "3")

    loocv_res = parallel::mclapply(seq_along(PIDS), FUN = function(i) {
        # Leave-one-out cross-validation split
        training_hiv = df_tuning %>% filter(PID != PIDS[i])
        testing_hiv  = df_tuning %>% filter(PID == PIDS[i])
        training_hiv_pl = dfpvl_tuning %>% filter(PID != PIDS[i])
        testing_hiv_pl  = dfpvl_tuning %>% filter(PID == PIDS[i])
        
        ## for intact DNA analysis
        fit_Intact = gam(
            as.formula(sub("viral_reservoir", "Intact", model_formula3)),
            data = training_hiv,
            method = "REML"
        )
        
        ## for defective DNA analysis
        fit_Defective = gam(
            as.formula(sub("viral_reservoir", "Defective", model_formula3)),
            data = training_hiv,
            method = "REML"
        )
        
        ## for defective DNA analysis
        fit_PlasmaVL = gam(
            as.formula(sub("viral_reservoir", "PlasmaVL", model_formula3_pl)),
            data = training_hiv_pl,
            method = "REML"
        )
        
        list(
            "predictionMAE_Intact" = mean(abs(predict(fit_Intact, newdata = testing_hiv) - log2(testing_hiv$Intact)), na.rm = TRUE),
            "predictionMSE_Intact" = mean((predict(fit_Intact, newdata = testing_hiv) - log2(testing_hiv$Intact))^2, na.rm = TRUE),
            "predictionMAE_Defective" = mean(abs(predict(fit_Defective, newdata = testing_hiv) - log2(testing_hiv$Defective)), na.rm = TRUE),
            "predictionMSE_Defective" = mean((predict(fit_Defective, newdata = testing_hiv) - log2(testing_hiv$Defective))^2, na.rm = TRUE),
            "predictionMAE_PlasmaVL" = mean(abs(predict(fit_PlasmaVL, newdata = testing_hiv_pl) - log2(testing_hiv_pl$PlasmaVL)), na.rm = TRUE),
            "predictionMSE_PlasmaVL" = mean((predict(fit_PlasmaVL, newdata = testing_hiv_pl) - log2(testing_hiv_pl$PlasmaVL))^2, na.rm = TRUE)
        )
    }, mc.cores = ncores)

    # collect results
    keys = names(loocv_res[[1]])
    loocv_res = do.call(mapply, c(FUN=c, lapply(loocv_res, `[`, keys)))

    # fit full models for AIC
    fit_Intact = gam(
        as.formula(sub("viral_reservoir", "Intact", model_formula)),
        data = df_tuning,
        method = "REML"
    )
    
    fit_Defective = gam(
        as.formula(sub("viral_reservoir", "Defective", model_formula)),
        data = df_tuning,
        method = "REML"
    )
    
    fit_PlasmaVL = gam(
        as.formula(sub("viral_reservoir", "PlasmaVL", model_formula_pl)),
        data = dfpvl_tuning,
        method = "REML"
    )

    # summarize results
    tibble(predictionMAE_Intact = mean(loocv_res[,"predictionMAE_Intact"], na.rm = TRUE),
           predictionMAEsd_Intact = sd(loocv_res[,"predictionMAE_Intact"], na.rm = TRUE),
           predictionMSE_Intact = mean(loocv_res[,"predictionMSE_Intact"], na.rm = TRUE),
           predictionMSEsd_Intact = sd(loocv_res[,"predictionMSE_Intact"], na.rm = TRUE),
           AIC_Intact = AIC(fit_Intact),
           adjR2_Intact = summary(fit_Intact)$r.sq,
           devExp_Intact = summary(fit_Intact)$dev.expl,
           predictionMAE_Defective = mean(loocv_res[,"predictionMAE_Defective"], na.rm = TRUE),
           predictionMAEsd_Defective = sd(loocv_res[,"predictionMAE_Defective"], na.rm = TRUE),
           predictionMSE_Defective = mean(loocv_res[,"predictionMSE_Defective"], na.rm = TRUE),
           predictionMSEsd_Defective = sd(loocv_res[,"predictionMSE_Defective"], na.rm = TRUE),
           AIC_Defective = AIC(fit_Defective),
           adjR2_Defective = summary(fit_Defective)$r.sq,
           devExp_Defective = summary(fit_Defective)$dev.expl,
           predictionMAE_PlasmaVL = mean(loocv_res[,"predictionMAE_PlasmaVL"], na.rm = TRUE),
           predictionMAEsd_PlasmaVL = sd(loocv_res[,"predictionMAE_PlasmaVL"], na.rm = TRUE),
           predictionMSE_PlasmaVL = mean(loocv_res[,"predictionMSE_PlasmaVL"], na.rm = TRUE),
           predictionMSEsd_PlasmaVL = sd(loocv_res[,"predictionMSE_PlasmaVL"], na.rm = TRUE),
           AIC_PlasmaVL = AIC(fit_PlasmaVL),
           adjR2_PlasmaVL = summary(fit_PlasmaVL)$r.sq,
           devExp_PlasmaVL = summary(fit_PlasmaVL)$dev.expl) %>%
        pivot_longer(cols = everything()) %>%
        separate(name, sep = "_", into = c("metric", "assay"))
}

expand_grid(tau1 = (0:54)/2, tau2 = (0:54)/2) %>% 
    filter(tau1 < tau2) %>%
    reframe(gam_tuning_loocv_3phase(tau1, tau2, dat_ipda = df, dat_pl = dfpvl, ncores = NCORES), .by = c(tau1, tau2)) %>%
    write_csv("loocv_tuning_triphasic.csv")
```

```
read_csv("loocv_tuning_triphasic.csv") %>%
    filter(tau1 < 25 & tau2 < 25) %>%
    filter(metric == "predictionMAE") %>%
    slice_min(value, by = assay) %>%
    kable()
```

| tau1 | tau2 | metric | assay | value |
| --- | --- | --- | --- | --- |
| 6.5 | 7.5 | predictionMAE | Intact | 2.140107 |
| 0.5 | 6.5 | predictionMAE | Defective | 1.680530 |
| 0.5 | 4.0 | predictionMAE | PlasmaVL | 1.516659 |

```
read_csv("loocv_tuning_triphasic.csv") %>%
    filter(tau1 < 25 & tau2 < 25) %>%
    filter(assay %in% c("Intact", "Defective")) %>%
    mutate(assay = factor(assay, levels = c("Intact", "Defective", "PlasmaVL"), labels = c("HIV Intact DNA", "HIV Defective DNA", "HIV Plasma RNA"))) %>%
    filter(metric %in% c("predictionMAE", "predictionMSE")) %>%
    mutate(metric = factor(metric, levels = c("predictionMAE", "predictionMSE"), labels = c("Prediction MAE", "Prediction MSE"))) %>%
    mutate(group = factor(paste(assay, metric, sep = ": "))) %>%
    mutate(group = factor(group, levels = levels(group)[c(3,1,4,2)])) %>%
    mutate(value = percent_rank(value), .by = c(metric, assay)) %>% # common scale: percentiles
    mutate(min = ifelse(value == min(value), " ", NA), .by = c(metric, assay)) %>%
    ggplot(aes(x = tau1, y = tau2, fill = value)) +
    facet_wrap(vars(group)) +
    geom_tile() + 
    geom_point(mapping = aes(color = min), data = . %>% filter(!is.na(min))) + 
    scale_x_continuous(name = expression('First Inflection Point' ~ tau[1] ~ '(Weeks on ART)'), breaks = (0:6)*4) +
    scale_y_continuous(name = expression('Second Inflection Point' ~ tau[2] ~ '(Weeks on ART)'), breaks = (0:6)*4) +
    scale_fill_continuous(name = "Scaled Loss Metric Rank", type = "viridis") + 
    scale_color_manual(name = "Loss Metric Minima", values = "red") +
    theme_pubr(base_size = 6, base_family = "Helvetica")
```

```
ggsave("figs/main/loocv_ipda_model_tuning_triphasic.pdf", width = 88, height = 100, units = "mm")


read_csv("loocv_tuning_triphasic.csv") %>%
    filter(tau1 < 25 & tau2 < 25) %>%
    filter(assay == "PlasmaVL") %>%
    mutate(assay = factor(assay, levels = c("Intact", "Defective", "PlasmaVL"), labels = c("HIV Intact DNA", "HIV Defective DNA", "HIV Plasma RNA"))) %>%
    filter(metric %in% c("predictionMAE", "predictionMSE")) %>%
    mutate(metric = factor(metric, levels = c("predictionMAE", "predictionMSE"), labels = c("Prediction MAE", "Prediction MSE"))) %>%
    mutate(group = factor(paste(assay, metric, sep = ": "))) %>%
    mutate(value = percent_rank(value), .by = c(metric, assay)) %>% # common scale: percentiles
    mutate(min = ifelse(value == min(value), " ", NA), .by = c(metric, assay)) %>%
    ggplot(aes(x = tau1, y = tau2, fill = value)) +
    facet_wrap(vars(group)) +
    geom_tile() +
    geom_point(mapping = aes(color = min), data = . %>% filter(!is.na(min))) + 
    scale_x_continuous(name = expression('First Inflection Point' ~ tau[1] ~ '(Weeks on ART)'), breaks = (0:6)*4) +
    scale_y_continuous(name = expression('Second Inflection Point' ~ tau[2] ~ '(Weeks on ART)'), breaks = (0:6)*4) +
    scale_fill_continuous(name = "Scaled Loss Metric Rank", type = "viridis") + 
    scale_color_manual(name = "Loss Metric Minima", values = "red", guide = guide_legend(direction = "horizontal")) +
    theme_pubr(base_size = 10, base_family = "Helvetica", legend = "right")
```

```
ggsave("figs/supp/loocv_pvl_model_tuning_triphasic.pdf", width = 6.5, height = 3, units = "in")
```

```
set.seed(1)

TAU_bi = read_csv("loocv_tuning_biphasic.csv") %>% 
    filter(metric == "predictionMAE") %>% 
    slice_min(value, by = "assay")

TAU_tri = read_csv("loocv_tuning_triphasic.csv") %>% 
    filter(metric == "predictionMAE") %>% 
    slice_min(value, by = "assay") %>%
    pivot_longer(starts_with("tau"), values_to = "tau")

bootstrap.AIC = function() {
    df_boot = df %>% 
        slice_sample(prop = 1, replace = TRUE) %>%
        select(-phase1, -phase2)

    dfpvl_boot = dfpvl %>% 
        slice_sample(prop = 1, replace = TRUE) %>%
        select(-phase1, -phase2, -phase3)
    
    mod_int_mono = gam(
        formula = as.formula(paste0(
            "log2(Intact) ~ ",
            "weeks_art_num + ",
            "weeks_art_num : eid_art_weeks + ",
            "s(log10firstvl, bs = 'cr', k = 20) + ",
            "s(first_cd4, bs = 'cr', k = 20) + ",
            "s(PID, bs = 're')")),
        family = gaussian(),
        data = df_boot,
        method = "REML"
    )
    
    mod_def_mono = gam(
        formula = as.formula(paste0(
            "log2(Defective) ~ ",
            "weeks_art_num + ",
            "weeks_art_num : eid_art_weeks + ",
            "s(log10firstvl, bs = 'cr', k = 20) + ",
            "s(first_cd4, bs = 'cr', k = 20) + ",
            "s(PID, bs = 're')")),
        family = gaussian(),
        data = df_boot,
        method = "REML"
    )
    
    mod_pvl_mono = gam(
        formula = as.formula(paste0(
            "log2(PlasmaVL) ~ ",
            "weeks_art_num + ",
            "weeks_art_num : eid_art_weeks + ",
            "s(first_cd4, bs = 'cr', k = 20) + ",
            "s(PID, bs = 're')")),
        family = gaussian(),
        data = dfpvl_boot,
        method = "REML"
    )
    
    mod_int_bi = gam(
        formula = as.formula(sub("viral_reservoir", "Intact", model_formula)),
        family = gaussian(),
        data = df_boot %>%
            bind_cols(lspline(.$weeks_art_num, knots = TAU_bi %>% filter(assay == "Intact") %>% pull(tau), marginal = FALSE)) %>% 
            rename(phase1 = "1", phase2 = "2"),
        method = "REML"
    )
    
    mod_def_bi = gam(
        formula = as.formula(sub("viral_reservoir", "Defective", model_formula)),
        family = gaussian(),
        data = df_boot %>%
            bind_cols(lspline(.$weeks_art_num, knots = TAU_bi %>% filter(assay == "Defective") %>% pull(tau), marginal = FALSE)) %>% 
            rename(phase1 = "1", phase2 = "2"),
        method = "REML"
    )

    mod_pvl_bi = gam(
        formula = as.formula(sub("viral_reservoir", "PlasmaVL", model_formula)),
        family = gaussian(),
        data = dfpvl_boot %>%
            bind_cols(lspline(.$weeks_art_num, knots = TAU_bi %>% filter(assay == "PlasmaVL") %>% pull(tau), marginal = FALSE)) %>% 
            rename(phase1 = "1", phase2 = "2"),
        method = "REML"
    )
    
    mod_int_tri = gam(
        as.formula(sub("viral_reservoir", "Intact", model_formula3)),
        data = df_boot %>%
            bind_cols(lspline(.$weeks_art_num, knots = TAU_tri %>% filter(assay == "Intact") %>% pull(tau), marginal = FALSE)) %>% 
            rename(phase1 = "1", phase2 = "2", phase3 = "3"),
        method = "REML"
    )
    
    mod_def_tri = gam(
        as.formula(sub("viral_reservoir", "Defective", model_formula3)),
        data = df_boot %>%
            bind_cols(lspline(.$weeks_art_num, knots = TAU_tri %>% filter(assay == "Defective") %>% pull(tau), marginal = FALSE)) %>% 
            rename(phase1 = "1", phase2 = "2", phase3 = "3"),
        method = "REML"
    )

    mod_pvl_tri = gam(
        as.formula(sub("viral_reservoir", "PlasmaVL", model_formula3)),
        data = dfpvl_boot %>%
            bind_cols(lspline(.$weeks_art_num, knots = TAU_tri %>% filter(assay == "Defective") %>% pull(tau), marginal = FALSE)) %>% 
            rename(phase1 = "1", phase2 = "2", phase3 = "3"),
        method = "REML"
    )
    
    tribble(
        ~reservoir,  ~model, ~AIC,
        "Intact",    "Mono", AIC(mod_int_mono),
        "Defective", "Mono", AIC(mod_def_mono),
        "PlasmaVL",  "Mono", AIC(mod_pvl_mono),
        "Intact",    "Bi",   AIC(mod_int_bi),
        "Defective", "Bi",   AIC(mod_def_bi),
        "PlasmaVL",  "Bi",   AIC(mod_pvl_bi),
        "Intact",    "Tri",  AIC(mod_int_tri),
        "Defective", "Tri",  AIC(mod_def_tri),
        "PlasmaVL",  "Tri",  AIC(mod_pvl_tri)
    )
}

tibble(iter = 1:500) %>%
    reframe(bootstrap.AIC(), .by = iter) %>%
    write_csv("bootstrap_AIC.csv")
```

```
read_csv("bootstrap_AIC.csv") %>%
    summarise(AIC_mean = mean(AIC),
              AIC_025 = quantile(AIC, probs = 0.025), 
              AIC_975 = quantile(AIC, probs = 0.975), .by = c(reservoir, model)) %>%
    mutate(value = paste0(round(AIC_mean,3), " (", round(AIC_025,3), ", ", round(AIC_975,3), ")")) %>%
    pivot_wider(id_cols = reservoir, names_from = model, values_from = value) %>%
    kable()
```

| reservoir | Mono | Bi | Tri |
| --- | --- | --- | --- |
| Intact | 884.019 (732.382, 1025.532) | 794.61 (590.176, 976.046) | 793.496 (589.581, 970.58) |
| Defective | 1425.679 (1341.859, 1499.293) | 1256.563 (1170.348, 1332.839) | 1253.884 (1167.6, 1334.553) |
| PlasmaVL | 2407.645 (2287.856, 2502.845) | 1927.971 (1807.54, 2026.12) | 1908.333 (1747.306, 2036.703) |

```
mod_pvl = gam(
    formula = as.formula(sub("viral_reservoir", "PlasmaVL", model_formula3_pl)),
    family = gaussian(),
    data = dfpvl,
    method = "REML"
)

dfpvl %>%
    summarise(eid_art_weeks = first(eid_art_weeks),
              log10firstvl = first(log10firstvl),
              first_cd4 = first(first_cd4),
              .by = PID) %>%
    select(-PID) %>%
    summarise(across(everything(), mean)) %>%
    expand_grid(weeks_art_num = (0:183) / 7) %>%
    mutate(PID = as.factor("a")) %>% # unknown/zero random effect
    bind_cols(lspline(.$weeks_art_num, knots = c(TAU1, TAU2), marginal = FALSE)) %>% 
    rename(phase1 = "1", phase2 = "2", phase3 = "3") %>% 
    mutate(PlasmaVL = 2^predict(mod_pvl, newdata = .)) %>%
    ggplot(aes(x = weeks_art_num, y = PlasmaVL)) +
    geom_line(mapping = aes(x = weeks_art_num, y = PlasmaVL, group = PID), 
              data = dfpvl, 
              color = "lightgrey", alpha = 0.5) +
    geom_line() +
    labs(x = "Sampling Timepoints (Weeks on ART)", 
         y = expression(atop("Plasma HIV RNA", "(copies / mL)"))) +
    scale_x_continuous(breaks = c(2,(0:6)*4)) +
    scale_y_log10() +
    theme_pubr()
```

```
# ggsave("figs/supp/predicted_average_patient_model_plasmavl.pdf", height = 4, width = 8)
```

```
plot(sm(getViz(mod_pvl), 1)) + 
    l_points(shape = 19, size = 1, alpha = 0.5) + 
    l_fitLine(colour = "red") + 
    l_ciLine(color = "blue") +
    l_rug(mapping = aes(x=x, y=y), alpha = 1) + 
    labs(x = expression("Initial CD4+ T Cell Count (Cells/"*mm^3*")"),
         y = expression(atop("Additive Effect to the", Log[2]*" Plasma HIV RNA"))) +
    theme_pubr(base_size = 11, base_family = "Helvetica")
```

```
ggsave("figs/supp/spline_terms_plasmavl.pdf", width = 3.5, height = 3, units = "in")
```

```
tibble(Assay = "Plasma HIV RNA", log2obs = mod_pvl$model$`log2(PlasmaVL)`, log2pred = fitted(mod_pvl)) %>%
    ggplot(aes(x = 2^(log2obs), y = 2^(log2pred))) + 
    facet_wrap(~Assay) +
    geom_point() + 
    geom_abline(slope = 1, intercept = 0, color = "red", linetype = "dashed") +
    xlab(expression("Observed Plasma HIV RNA (copies/mL)")) +
    ylab(expression("Predicted Plasma HIV RNA (copies/mL)")) +
    scale_x_log10() + 
    scale_y_log10() +
    theme_pubr(base_size = 10, base_family = "Helvetica")
```

```
ggsave("figs/supp/diagnostic_pred_vs_fitted_plasmavl.pdf", width = 3.5, height = 3, units = "in")
```

```
# create random patients, then predict their reservoir decays
set.seed(1)
df_sim = dfpvl %>%
    select(PID, eid_art_days, first_cd4) %>%
    distinct() %>%
    sample_n(size = 300, replace = TRUE) %>% # random rows
    mutate(across(everything(), sample)) # shuffle each column (break existing patient relationship)

ggarrange(
    df_sim %>% 
        mutate(tertile = ifelse(eid_art_days + avg_eid_art_days < 30, "<30 days", "30-100 days")) %>%
        expand_grid(weeks_art_num = (0:183) / 7) %>%
        mutate(eid_art_weeks = eid_art_days / 7) %>%
        bind_cols(lspline(.$weeks_art_num, knots = c(TAU1, TAU2), marginal = FALSE)) %>% 
        rename(phase1 = "1", phase2 = "2", phase3 = "3") %>% 
        mutate(PlasmaVL = as.numeric(predict(mod_pvl, .))) %>% # predict each patient's decay pattern
        mutate(PlasmaVL = 2^PlasmaVL) %>%
        ggplot(aes(x = weeks_art_num, y = PlasmaVL)) +
        geom_smooth(aes(color = tertile)) +
        labs(x = "Sampling Timepoints (Weeks on ART)", 
             y = expression("Plasma HIV RNA (copies / mL)"),
             color = "Timing of ART initiation") +
        scale_x_continuous(breaks = c(2,(0:6)*4)) +
        scale_y_log10() +
        theme_pubr(base_size = 11, base_family = "Helvetica", legend = "top"),
    df_sim %>% 
        arrange(first_cd4) %>% # sort rows/patients by variable of interest
        mutate(tertile = rep(c("Bottom", "Top"), each = n()/2)) %>% # identify tertiles
        filter(tertile != "Second") %>%
        mutate(LB = signif(min(first_cd4), digits = 2), UB = signif(max(first_cd4), digits = 2), .by = tertile) %>% # make pretty labels for tertiles
        mutate(tertile = factor(tertile, 
                                levels = c("Bottom", "Top"),
                                labels = unique(paste0(tertile, " Half (", LB, "-", UB, "]")))) %>%
        expand_grid(weeks_art_num = (0:183) / 7) %>%
        mutate(eid_art_weeks = eid_art_days / 7) %>%
        bind_cols(lspline(.$weeks_art_num, knots = c(TAU1, TAU2), marginal = FALSE)) %>% 
        rename(phase1 = "1", phase2 = "2", phase3 = "3") %>% 
        mutate(PlasmaVL = as.numeric(predict(mod_pvl, .))) %>% # predict each patient's decay pattern
        mutate(PlasmaVL = 2^PlasmaVL) %>%
        ggplot(aes(x = weeks_art_num, y = PlasmaVL)) +
        geom_smooth(aes(color = tertile)) +
        labs(x = "Sampling Timepoints (Weeks on ART)", 
             y = expression("Plasma HIV RNA (copies / mL)"),
             color = expression("Initial CD4 Count (Cells/"*mm^3*")")) +
        scale_x_continuous(breaks = c(2,(0:6)*4)) +
        scale_y_log10() +
        theme_pubr(base_size = 11, base_family = "Helvetica", legend = "top"),
    nrow = 2,
    labels = c("a.", "b.")
)
```

```
ggsave("figs/supp/simulated_predicted_plasmavl_decays.pdf", width = 6.5, height = 6, units = "in")
```

# Model Sensitivity

## Full Year Models

```
tibble(tau = (0:106)/2) %>%
    reframe(gam_tuning_loocv_2phase(tau, 
                                    dat_ipda = filter(df_full, days_art_num <= 365), 
                                    dat_pl = filter(dfpvl_full, days_art_num <= 365), 
                                    ncores = NCORES), .by = tau) %>%
    write_csv("loocv_tuning_biphasic_year.csv")

expand_grid(tau1 = (0:106)/2, tau2 = (0:106)/2) %>% 
    filter(tau1 < tau2) %>%
    reframe(gam_tuning_loocv_3phase(tau1, tau2, 
                                    dat_ipda = filter(df_full, days_art_num <= 365), 
                                    dat_pl = filter(dfpvl_full, days_art_num <= 365), 
                                    ncores = NCORES), .by = c(tau1, tau2)) %>%
    write_csv("loocv_tuning_triphasic_year.csv")
```

```
mod_int_year = gam(
    formula = as.formula(sub("viral_reservoir", "Intact", model_formula)),
    family = gaussian(),
    data = df_full %>% filter(days_art_num <= 365),
    method = "REML"
)

mod_def_year = gam(
    formula = as.formula(sub("viral_reservoir", "Defective", model_formula)),
    family = gaussian(),
    data = df_full %>% filter(days_art_num <= 365),
    method = "REML"
)

df_full %>%
    filter(days_art_num <= 365) %>%
    summarise(eid_art_weeks = first(eid_art_weeks),
              log10firstvl = first(log10firstvl),
              first_cd4 = first(first_cd4),
              .by = PID) %>%
    select(-PID) %>%
    summarise(across(everything(), mean)) %>%
    expand_grid(weeks_art_num = (0:365) / 7) %>%
    mutate(PID = as.factor("a")) %>% # unknown/zero random effect
    bind_cols(lspline(.$weeks_art_num, knots = TAU, marginal = FALSE)) %>% 
    rename(phase1 = "1", phase2 = "2") %>% 
    mutate(Intact = 2^predict(mod_int_year, newdata = .)) %>%
    mutate(Defective = 2^predict(mod_def_year, newdata = .)) %>%
    pivot_longer(cols = c(Intact, Defective)) %>%
    mutate(name = factor(name, levels = c("Intact", "Defective"), labels = c("HIV Intact HIV", "HIV Defective HIV"))) %>%
    ggplot(aes(x = weeks_art_num, y = value)) +
    facet_wrap(~name, scales = "free_y") +
    geom_line(mapping = aes(x = weeks_art_num, y = value, group = PID), 
              data = df_full %>%
                  filter(days_art_num <= 365) %>% 
                  pivot_longer(c(Intact, Defective)) %>% 
                  mutate(name = factor(name, levels = c("Intact", "Defective"), labels = c("HIV Intact HIV", "HIV Defective HIV"))), 
              color = "lightgrey", alpha = 0.5) +
    geom_line() +
    scale_x_continuous(name = "Sampling Timepoints (Weeks on ART)",
                       breaks = c(2,(0:6)*4, (3:4)*12), 
                       labels = ~if_else(near(.x %% 8, 0), as.character(.x), "")) +
    scale_y_log10(name = expression(atop("HIV DNA", "(copies /" * 10^6 * " CD4+ T Cells)"))) +
    theme_pubr(base_size = 11, base_family = "Helvetica")
```

```
ggsave("figs/supp/predicted_average_patient_model_main_year.pdf", width = 6.5, height = 3, units = "in")
```

```
read_csv("loocv_tuning_biphasic_year.csv") %>%
    filter(tau <= 46) %>%
    filter(metric %in% c("predictionMAE", "predictionMSE")) %>%
    mutate(metric = factor(metric, levels = c("predictionMAE", "predictionMSE"), labels = c("Prediction MAE", "Prediction MSE"))) %>%
    filter(assay %in% c("Intact", "Defective")) %>%
    mutate(assay = factor(assay, levels = c("Intact", "Defective", "PlasmaVL"), labels = c("HIV Intact DNA", "HIV Defective DNA", "HIV Plasma RNA"))) %>%
    mutate(group = factor(paste(assay, metric, sep = ": "))) %>%
    mutate(group = factor(group, levels = levels(group)[c(3,1,4,2)])) %>%
    mutate(ismin = value == min(value), .by = c(metric, assay)) %>%
    ggplot(data = ., aes(x = tau, y = value, color = ismin)) +
    facet_wrap(vars(group), scales = "free_y") +
    geom_point() + 
    geom_vline(xintercept = TAU, linetype = "dashed") +
    scale_x_continuous(name = expression(paste("Inflection Point" ~ tau ~ "(Weeks on ART)"))) +
    scale_y_continuous(name = "Loss Metric") +
    scale_color_manual(name = "Loss Metric Minima", values = c("TRUE" = "red", "FALSE" = "black")) +
    theme_pubr(base_size = 11, base_family = "Helvetica", ) +
    theme(legend.box.spacing = unit(0, "mm"))
```

```
ggsave("figs/supp/loocv_model_tuning_year.pdf", width = 6.5, height = 5, units = "in")
```

## Drop Participants with PrEP use

```
tibble(tau = (0:52)/2) %>%
    reframe(gam_tuning_loocv_2phase(tau, 
                                    dat_ipda = filter(df, prep_group == 0), 
                                    dat_pl = filter(dfpvl, prep_group == 0), 
                                    ncores = NCORES), .by = tau) %>%
    write_csv("loocv_tuning_biphasic_noPrEP.csv")

expand_grid(tau1 = (0:52)/2, tau2 = (0:52)/2) %>% 
    filter(tau1 < tau2) %>%
    reframe(gam_tuning_loocv_3phase(tau1, tau2, 
                                    dat_ipda = filter(df, prep_group == 0), 
                                    dat_pl = filter(dfpvl, prep_group == 0), 
                                    ncores = NCORES), .by = c(tau1, tau2)) %>%
    write_csv("loocv_tuning_triphasic_noPrEP.csv")
```

```
mod_int_noprep = gam(
    formula = as.formula(sub("viral_reservoir", "Intact", model_formula)),
    family = gaussian(),
    data = filter(df, prep_group == 0),
    method = "REML"
)

mod_def_noprep = gam(
    formula = as.formula(sub("viral_reservoir", "Defective", model_formula)),
    family = gaussian(),
    data = filter(df, prep_group == 0),
    method = "REML"
)
```

## Drop Participants with Plasma HIV Viral Load Blips

```
dfpvl_full %>%
    filter(weeks_art_num > 4, weeks_art_num < 52) %>% 
    arrange(PID, days_art_num) %>% 
    filter(PlasmaVL > 40) %>%
    select(PID, days_art_num, PlasmaVL)
```

```
## # A tibble: 39 × 3
##    PID                                  days_art_num PlasmaVL
##    <fct>                                       <dbl>    <dbl>
##  1 00869785-e8d5-4bf2-a93a-22b2ffef91cc          112       41
##  2 0727f593-30d5-4a26-95d9-7a854cb2b08d           42      422
##  3 0727f593-30d5-4a26-95d9-7a854cb2b08d           56      380
##  4 0727f593-30d5-4a26-95d9-7a854cb2b08d           97      262
##  5 0727f593-30d5-4a26-95d9-7a854cb2b08d          112      165
##  6 0727f593-30d5-4a26-95d9-7a854cb2b08d          147       85
##  7 0727f593-30d5-4a26-95d9-7a854cb2b08d          167      153
##  8 0727f593-30d5-4a26-95d9-7a854cb2b08d          201       97
##  9 0727f593-30d5-4a26-95d9-7a854cb2b08d          260      120
## 10 07fad2aa-bfba-4075-bf62-97fadda760ae           29      146
## # ℹ 29 more rows
```

```
blip_ids = c( # at least one blip over 1000 or two consecutive blips above ~100
    "9e5d1f5a-8872-40d4-810c-4eba914b185f",
    "9521f44f-ea36-481c-a853-6b8e57f30501",
    "1a69a256-1ccb-4e64-9013-3e7eade34283",
    "0727f593-30d5-4a26-95d9-7a854cb2b08d"
)
```

```
tibble(tau = (0:52)/2) %>%
    reframe(gam_tuning_loocv_2phase(tau, 
                                    dat_ipda = filter(df, !(PID %in% blip_ids)), 
                                    dat_pl = filter(dfpvl, !(PID %in% blip_ids)), 
                                    ncores = NCORES), .by = tau) %>%
    write_csv("loocv_tuning_biphasic_noBlip.csv")

expand_grid(tau1 = (0:52)/2, tau2 = (0:52)/2) %>% 
    filter(tau1 < tau2) %>%
    reframe(gam_tuning_loocv_3phase(tau1, tau2, 
                                    dat_ipda = filter(df, !(PID %in% blip_ids)), 
                                    dat_pl = filter(dfpvl, !(PID %in% blip_ids)), 
                                    ncores = NCORES), .by = c(tau1, tau2)) %>%
    write_csv("loocv_tuning_triphasic_noBlip.csv")
```

```
mod_int_noblip = gam(
    formula = as.formula(sub("viral_reservoir", "Intact", model_formula)),
    family = gaussian(),
    data = filter(df, !(PID %in% blip_ids)),
    method = "REML"
)

mod_def_noblip = gam(
    formula = as.formula(sub("viral_reservoir", "Defective", model_formula)),
    family = gaussian(),
    data = filter(df, !(PID %in% blip_ids)),
    method = "REML"
)
```

## Drop Participants with Increasing Intact HIV Viral Reservoir

```
intact_filtered_ids = setdiff( # people dropped
    as.character(df$PID), 
    df %>% 
    arrange(PID, days_art_num) %>% 
    mutate(delta_intact = c(0, diff(Intact))) %>% 
    filter(!any(delta_intact > 0.5*Intact & weeks_art_num > 4), .by = PID) %>%
    pull(PID) %>% 
    as.character()
)
```

```
tibble(tau = (0:52)/2) %>%
    reframe(gam_tuning_loocv_2phase(tau, 
                                    dat_ipda = filter(df, !(PID %in% intact_filtered_ids)), 
                                    dat_pl = filter(dfpvl, !(PID %in% intact_filtered_ids)), 
                                    ncores = NCORES), .by = tau) %>%
    write_csv("loocv_tuning_biphasic_IntactIncr.csv")

expand_grid(tau1 = (0:52)/2, tau2 = (0:52)/2) %>% 
    filter(tau1 < tau2) %>%
    reframe(gam_tuning_loocv_3phase(tau1, tau2, 
                                    dat_ipda = filter(df, !(PID %in% intact_filtered_ids)), 
                                    dat_pl = filter(dfpvl, !(PID %in% intact_filtered_ids)), 
                                    ncores = NCORES), .by = c(tau1, tau2)) %>%
    write_csv("loocv_tuning_triphasic_IntactIncr.csv")
```

```
mod_int_inc = gam(
    formula = as.formula(sub("viral_reservoir", "Intact", model_formula)),
    family = gaussian(),
    data = filter(df, !(PID %in% intact_filtered_ids)),
    method = "REML"
)

mod_def_inc = gam(
    formula = as.formula(sub("viral_reservoir", "Defective", model_formula)),
    family = gaussian(),
    data = filter(df, !(PID %in% intact_filtered_ids)),
    method = "REML"
)
```

## Combined Figures

```
ggarrange(
    df %>%
        filter(prep_group == 0) %>%
        summarise(eid_art_weeks = first(eid_art_weeks),
                  log10firstvl = first(log10firstvl),
                  first_cd4 = first(first_cd4),
                  .by = PID) %>%
        select(-PID) %>%
        summarise(across(everything(), mean)) %>%
        expand_grid(weeks_art_num = (0:183) / 7) %>%
        mutate(PID = as.factor("a")) %>% # unknown/zero random effect
        bind_cols(lspline(.$weeks_art_num, knots = TAU, marginal = FALSE)) %>% 
        rename(phase1 = "1", phase2 = "2") %>% 
        mutate(Intact = 2^predict(mod_int_noprep, newdata = .)) %>%
        mutate(Defective = 2^predict(mod_def_noprep, newdata = .)) %>%
        pivot_longer(cols = c(Intact, Defective)) %>%
        mutate(name = factor(name, levels = c("Intact", "Defective"), labels = c("HIV Intact DNA", "HIV Defective DNA"))) %>%
        ggplot(aes(x = weeks_art_num, y = value, color = "Predicted Average")) +
        facet_wrap(~name, scales = "free_y") +
        geom_line(mapping = aes(x = weeks_art_num, y = value, group = PID, color = "Excluded"), 
                  data = df %>% 
                      filter(prep_group != 0) %>%
                      pivot_longer(c(Intact, Defective)) %>% 
                      mutate(name = factor(name, levels = c("Intact", "Defective"), labels = c("HIV Intact DNA", "HIV Defective DNA"))), 
                  alpha = 1) +
        geom_line(mapping = aes(x = weeks_art_num, y = value, group = PID, color = "Included"), 
                  data = df %>% 
                      filter(prep_group == 0) %>%
                      pivot_longer(c(Intact, Defective)) %>% 
                      mutate(name = factor(name, levels = c("Intact", "Defective"), labels = c("HIV Intact DNA", "HIV Defective DNA"))), 
                  alpha = 0.5) +
        geom_line() +
        scale_x_continuous(name = "Sampling Timepoints (Weeks on ART)", breaks = c(2,(0:6)*4)) +
        scale_y_log10(name = expression(atop("HIV DNA", "(copies /" * 10^6 * " CD4+ T Cells)"))) +
        scale_color_manual(name = "Participant", values = c("Excluded" = "red", "Included" = "lightgrey", "Predicted Average" = "black")) +
        theme_pubr(base_size = 11, base_family = "Helvetica"),
    df %>%
        filter(prep_group == 0) %>%
        summarise(eid_art_weeks = first(eid_art_weeks),
                  log10firstvl = first(log10firstvl),
                  first_cd4 = first(first_cd4),
                  .by = PID) %>%
        select(-PID) %>%
        summarise(across(everything(), mean)) %>%
        expand_grid(weeks_art_num = (0:183) / 7) %>%
        mutate(PID = as.factor("a")) %>% # unknown/zero random effect
        bind_cols(lspline(.$weeks_art_num, knots = TAU, marginal = FALSE)) %>% 
        rename(phase1 = "1", phase2 = "2") %>% 
        mutate(Intact = 2^predict(mod_int_noblip, newdata = .)) %>%
        mutate(Defective = 2^predict(mod_def_noblip, newdata = .)) %>%
        pivot_longer(cols = c(Intact, Defective)) %>%
        mutate(name = factor(name, levels = c("Intact", "Defective"), labels = c("HIV Intact DNA", "HIV Defective DNA"))) %>%
        ggplot(aes(x = weeks_art_num, y = value, color = "Predicted Average")) +
        facet_wrap(~name, scales = "free_y") +
        geom_line(mapping = aes(x = weeks_art_num, y = value, group = PID, color = "Excluded"), 
                  data = df %>% 
                      filter(PID %in% blip_ids) %>%
                      pivot_longer(c(Intact, Defective)) %>% 
                      mutate(name = factor(name, levels = c("Intact", "Defective"), labels = c("HIV Intact DNA", "HIV Defective DNA"))), 
                  alpha = 1) +
        geom_line(mapping = aes(x = weeks_art_num, y = value, group = PID, color = "Included"), 
                  data = df %>% 
                      filter(!(PID %in% blip_ids)) %>%
                      pivot_longer(c(Intact, Defective)) %>% 
                      mutate(name = factor(name, levels = c("Intact", "Defective"), labels = c("HIV Intact DNA", "HIV Defective DNA"))), 
                  alpha = 0.5) +
        geom_line() +
        scale_x_continuous(name = "Sampling Timepoints (Weeks on ART)", breaks = c(2,(0:6)*4)) +
        scale_y_log10(name = expression(atop("HIV DNA", "(copies /" * 10^6 * " CD4+ T Cells)"))) +
        scale_color_manual(name = "Participant", values = c("Excluded" = "red", "Included" = "lightgrey", "Predicted Average" = "black")) +
        theme_pubr(base_size = 11, base_family = "Helvetica"),
    df %>%
        filter(prep_group == 0) %>%
        summarise(eid_art_weeks = first(eid_art_weeks),
                  log10firstvl = first(log10firstvl),
                  first_cd4 = first(first_cd4),
                  .by = PID) %>%
        select(-PID) %>%
        summarise(across(everything(), mean)) %>%
        expand_grid(weeks_art_num = (0:183) / 7) %>%
        mutate(PID = as.factor("a")) %>% # unknown/zero random effect
        bind_cols(lspline(.$weeks_art_num, knots = TAU, marginal = FALSE)) %>% 
        rename(phase1 = "1", phase2 = "2") %>% 
        mutate(Intact = 2^predict(mod_int_inc, newdata = .)) %>%
        mutate(Defective = 2^predict(mod_def_inc, newdata = .)) %>%
        pivot_longer(cols = c(Intact, Defective)) %>%
        mutate(name = factor(name, levels = c("Intact", "Defective"), labels = c("HIV Intact DNA", "HIV Defective DNA"))) %>%
        ggplot(aes(x = weeks_art_num, y = value, color = "Predicted Average")) +
        facet_wrap(~name, scales = "free_y") +
        geom_line(mapping = aes(x = weeks_art_num, y = value, group = PID, color = "Excluded"), 
                  data = df %>% 
                      filter(PID %in% intact_filtered_ids) %>%
                      pivot_longer(c(Intact, Defective)) %>% 
                      mutate(name = factor(name, levels = c("Intact", "Defective"), labels = c("HIV Intact DNA", "HIV Defective DNA"))), 
                  alpha = 1) +
        geom_line(mapping = aes(x = weeks_art_num, y = value, group = PID, color = "Included"), 
                  data = df %>% 
                      filter(!(PID %in% intact_filtered_ids)) %>%
                      pivot_longer(c(Intact, Defective)) %>% 
                      mutate(name = factor(name, levels = c("Intact", "Defective"), labels = c("HIV Intact DNA", "HIV Defective DNA"))), 
                  alpha = 0.5) +
        geom_line() +
        scale_x_continuous(name = "Sampling Timepoints (Weeks on ART)", breaks = c(2,(0:6)*4)) +
        scale_y_log10(name = expression(atop("HIV DNA", "(copies /" * 10^6 * " CD4+ T Cells)"))) +
        scale_color_manual(name = "Participant", values = c("Excluded" = "red", "Included" = "lightgrey", "Predicted Average" = "black")) +
        theme_pubr(base_size = 11, base_family = "Helvetica"),
    nrow = 3,
    labels = c("a.", "b.", "c.")
)
```

```
ggsave("figs/supp/biphasic_model_sensitivity.pdf", width = 6.5, height = 9, units = "in")
```

```
ggarrange(
    read_csv("loocv_tuning_biphasic_noPrEP.csv") %>%
        filter(assay %in% c("Intact", "Defective")) %>%
        filter(metric == "predictionMAE") %>%
        filter(tau <= 25) %>%
        mutate(assay = factor(assay, levels = c("Intact", "Defective"), labels = c("HIV Intact DNA", "HIV Defective DNA"))) %>%
        mutate(ismin = value == min(value), .by = c(metric, assay)) %>%
        ggplot(aes(x = tau, y = value, color = ismin)) +
        facet_wrap(~ assay, scales = "free_y") +
        geom_point() +
        geom_vline(xintercept = TAU, linetype = "dashed") +
        scale_x_continuous(name = expression(paste("Inflection Point ", tau, " (Weeks on ART)")), breaks = (0:6)*4) +
        scale_y_continuous(name = "LOOCV Mean Prediction Absolute Error") +
        scale_color_manual(name = "Loss Metric Minima", values = c("TRUE" = "red", "FALSE" = "black")) +
        theme_pubr(base_size = 10, base_family = "Helvetica"),
    read_csv("loocv_tuning_biphasic_noBlip.csv") %>%
        filter(assay %in% c("Intact", "Defective")) %>%
        filter(metric == "predictionMAE") %>%
        filter(tau <= 25) %>%
        mutate(assay = factor(assay, levels = c("Intact", "Defective"), labels = c("HIV Intact DNA", "HIV Defective DNA"))) %>%
        mutate(ismin = value == min(value), .by = c(metric, assay)) %>%
        ggplot(aes(x = tau, y = value, color = ismin)) +
        facet_wrap(~ assay, scales = "free_y") +
        geom_point() +
        geom_vline(xintercept = TAU, linetype = "dashed") +
        scale_x_continuous(name = expression(paste("Inflection Point ", tau, " (Weeks on ART)")), breaks = (0:6)*4) +
        scale_y_continuous(name = "LOOCV Mean Prediction Absolute Error") +
        scale_color_manual(name = "Loss Metric Minima", values = c("TRUE" = "red", "FALSE" = "black")) +
        theme_pubr(base_size = 10, base_family = "Helvetica"),
    read_csv("loocv_tuning_biphasic_IntactIncr.csv") %>%
        filter(assay %in% c("Intact", "Defective")) %>%
        filter(metric == "predictionMAE") %>%
        filter(tau <= 25) %>%
        mutate(assay = factor(assay, levels = c("Intact", "Defective"), labels = c("HIV Intact DNA", "HIV Defective DNA"))) %>%
        mutate(ismin = value == min(value), .by = c(metric, assay)) %>%
        ggplot(aes(x = tau, y = value, color = ismin)) +
        facet_wrap(~ assay, scales = "free_y") +
        geom_point() +
        geom_vline(xintercept = TAU, linetype = "dashed") +
        scale_x_continuous(name = expression(paste("Inflection Point ", tau, " (Weeks on ART)")), breaks = (0:6)*4) +
        scale_y_continuous(name = "LOOCV Mean Prediction Absolute Error") +
        scale_color_manual(name = "Loss Metric Minima", values = c("TRUE" = "red", "FALSE" = "black")) +
        theme_pubr(base_size = 10, base_family = "Helvetica"),
    nrow = 3,
    labels = c("a.", "b.", "c.")
)
```

```
ggsave("figs/supp/biphasic_tuning_sensitivity.pdf", width = 6.5, height = 9, units = "in")
```

# Model Estimates and Fits

```
mod_int_ua = gam(
    formula = as.formula(sub("viral_reservoir", "Intact", model_formula_unadjusted)),
    family = gaussian(),
    data = df,
    method = "REML"
)

mod_def_ua = gam(
    formula = as.formula(sub("viral_reservoir", "Defective", model_formula_unadjusted)),
    family = gaussian(),
    data = df,
    method = "REML"
)

mod_int_ua_year = gam(
    formula = as.formula(sub("viral_reservoir", "Intact", model_formula_unadjusted)),
    family = gaussian(),
    data = filter(df_full, days_art_num <= 365),
    method = "REML"
)

mod_def_ua_year = gam(
    formula = as.formula(sub("viral_reservoir", "Defective", model_formula_unadjusted)),
    family = gaussian(),
    data = filter(df_full, days_art_num <= 365),
    method = "REML"
)

tribble(
    ~Model,      ~Outcome,     ~Phase,                       ~N_participants,                              ~Estimate,                                    ~SE,                                      ~p_value,
    "Week 0-24", "Intact",     paste0("0-", TAU, " Weeks"),  length(unique(mod_int_ua$model$PID)),         summary(mod_int_ua)$p.coef[["phase1"]],       summary(mod_int_ua)$se[["phase1"]],       summary(mod_int_ua)$p.pv[["phase1"]],
    "Week 0-24", "Defective",  paste0("0-", TAU, " Weeks"),  length(unique(mod_def_ua$model$PID)),         summary(mod_def_ua)$p.coef[["phase1"]],       summary(mod_def_ua)$se[["phase1"]],       summary(mod_def_ua)$p.pv[["phase1"]],
    "Week 0-24", "Intact",     paste0(TAU, "-24 Weeks"),     length(unique(mod_int_ua$model$PID)),         summary(mod_int_ua)$p.coef[["phase2"]],       summary(mod_int_ua)$se[["phase2"]],       summary(mod_int_ua)$p.pv[["phase2"]],
    "Week 0-24", "Defective",  paste0(TAU, "-24 Weeks"),     length(unique(mod_def_ua$model$PID)),         summary(mod_def_ua)$p.coef[["phase2"]],       summary(mod_def_ua)$se[["phase2"]],       summary(mod_def_ua)$p.pv[["phase2"]],

    "Week 0-52", "Intact",       paste0("0-", TAU, " Weeks"),  length(unique(mod_int_ua_year$model$PID)),  summary(mod_int_ua_year)$p.coef[["phase1"]],  summary(mod_int_ua_year)$se[["phase1"]],  summary(mod_int_ua_year)$p.pv[["phase1"]],
    "Week 0-52", "Defective",    paste0("0-", TAU, " Weeks"),  length(unique(mod_def_ua_year$model$PID)),  summary(mod_def_ua_year)$p.coef[["phase1"]],  summary(mod_def_ua_year)$se[["phase1"]],  summary(mod_def_ua_year)$p.pv[["phase1"]],
    "Week 0-52", "Intact",       paste0(TAU, "-52 Weeks"),     length(unique(mod_int_ua_year$model$PID)),  summary(mod_int_ua_year)$p.coef[["phase2"]],  summary(mod_int_ua_year)$se[["phase2"]],  summary(mod_int_ua_year)$p.pv[["phase2"]],
    "Week 0-52", "Defective",    paste0(TAU, "-52 Weeks"),     length(unique(mod_def_ua_year$model$PID)),  summary(mod_def_ua_year)$p.coef[["phase2"]],  summary(mod_def_ua_year)$se[["phase2"]],  summary(mod_def_ua_year)$p.pv[["phase2"]],
    ) %>%
    mutate(PercentDecayRatePerWeek = -100*(2^Estimate - 1)) %>% # negative because it's in terms of decay rather than percent change
    mutate(halflife_week = -1/Estimate) %>%
    mutate(halflifesd = 1/Estimate^2*SE) %>%
    mutate(halflife_lowCI = halflife_week - 1.96*halflifesd, halflife_highCI = halflife_week + 1.96*halflifesd) %>%
    select(-halflifesd) %>% 
    kable()
```

| Model | Outcome | Phase | N\_participants | Estimate | SE | p\_value | PercentDecayRatePerWeek | halflife\_week | halflife\_lowCI | halflife\_highCI |
| --- | --- | --- | --- | --- | --- | --- | --- | --- | --- | --- |
| Week 0-24 | Intact | 0-5 Weeks | 61 | -0.3565870 | 0.0297234 | 0.0000000 | 21.8989946 | 2.804365 | 2.346198 | 3.262531 |
| Week 0-24 | Defective | 0-5 Weeks | 61 | -0.7445326 | 0.0548906 | 0.0000000 | 40.3138800 | 1.343125 | 1.149042 | 1.537207 |
| Week 0-24 | Intact | 5-24 Weeks | 61 | -0.0691860 | 0.0078421 | 0.0000000 | 4.6824344 | 14.453793 | 11.242705 | 17.664882 |
| Week 0-24 | Defective | 5-24 Weeks | 61 | 0.0018380 | 0.0145115 | 0.8992956 | -0.1274787 | -544.082001 | -8963.799265 | 7875.635263 |
| Week 0-52 | Intact | 0-5 Weeks | 61 | -0.3837819 | 0.0280254 | 0.0000000 | 23.3574159 | 2.605647 | 2.232707 | 2.978586 |
| Week 0-52 | Defective | 0-5 Weeks | 61 | -0.7510224 | 0.0512198 | 0.0000000 | 40.5817678 | 1.331518 | 1.153531 | 1.509505 |
| Week 0-52 | Intact | 5-52 Weeks | 61 | -0.0538688 | 0.0049780 | 0.0000000 | 3.6650472 | 18.563637 | 15.201327 | 21.925947 |
| Week 0-52 | Defective | 5-52 Weeks | 61 | 0.0102196 | 0.0091035 | 0.2623999 | -0.7108837 | -97.851173 | -268.694405 | 72.992059 |

```
tribble(
    ~Model,      ~Outcome,       ~Phase,                       ~N_participants,                           ~Estimate,                                   ~SE,                                     ~p_value,
    "Week 0-24", "Intact",       paste0("0-", TAU, " Weeks"),  length(unique(mod_int$model$PID)),         summary(mod_int)$p.coef[["phase1"]],         summary(mod_int)$se[["phase1"]],         summary(mod_int)$p.pv[["phase1"]],
    "Week 0-24", "Defective",    paste0("0-", TAU, " Weeks"),  length(unique(mod_def$model$PID)),         summary(mod_def)$p.coef[["phase1"]],         summary(mod_def)$se[["phase1"]],         summary(mod_def)$p.pv[["phase1"]],
    "Week 0-24", "Intact",       paste0(TAU, "-24 Weeks"),     length(unique(mod_int$model$PID)),         summary(mod_int)$p.coef[["phase2"]],         summary(mod_int)$se[["phase2"]],         summary(mod_int)$p.pv[["phase2"]],
    "Week 0-24", "Defective",    paste0(TAU, "-24 Weeks"),     length(unique(mod_def$model$PID)),         summary(mod_def)$p.coef[["phase2"]],         summary(mod_def)$se[["phase2"]],         summary(mod_def)$p.pv[["phase2"]],

    "Week 0-52", "Intact",       paste0("0-", TAU, " Weeks"),  length(unique(mod_int_year$model$PID)),    summary(mod_int_year)$p.coef[["phase1"]],    summary(mod_int_year)$se[["phase1"]],    summary(mod_int_year)$p.pv[["phase1"]],
    "Week 0-52", "Defective",    paste0("0-", TAU, " Weeks"),  length(unique(mod_def_year$model$PID)),    summary(mod_def_year)$p.coef[["phase1"]],    summary(mod_def_year)$se[["phase1"]],    summary(mod_def_year)$p.pv[["phase1"]],
    "Week 0-52", "Intact",       paste0(TAU, "-24 Weeks"),     length(unique(mod_int_year$model$PID)),    summary(mod_int_year)$p.coef[["phase2"]],    summary(mod_int_year)$se[["phase2"]],    summary(mod_int_year)$p.pv[["phase2"]],
    "Week 0-52", "Defective",    paste0(TAU, "-24 Weeks"),     length(unique(mod_def_year$model$PID)),    summary(mod_def_year)$p.coef[["phase2"]],    summary(mod_def_year)$se[["phase2"]],    summary(mod_def_year)$p.pv[["phase2"]],

    "Drop PrEP", "Intact",       paste0("0-", TAU, " Weeks"),  length(unique(mod_int_noprep$model$PID)),  summary(mod_int_noprep)$p.coef[["phase1"]],  summary(mod_int_noprep)$se[["phase1"]],  summary(mod_int_noprep)$p.pv[["phase1"]],
    "Drop PrEP", "Defective",    paste0("0-", TAU, " Weeks"),  length(unique(mod_def_noprep$model$PID)),  summary(mod_def_noprep)$p.coef[["phase1"]],  summary(mod_def_noprep)$se[["phase1"]],  summary(mod_def_noprep)$p.pv[["phase1"]],
    "Drop PrEP", "Intact",       paste0(TAU, "-24 Weeks"),     length(unique(mod_int_noprep$model$PID)),  summary(mod_int_noprep)$p.coef[["phase2"]],  summary(mod_int_noprep)$se[["phase2"]],  summary(mod_int_noprep)$p.pv[["phase2"]],
    "Drop PrEP", "Defective",    paste0(TAU, "-24 Weeks"),     length(unique(mod_def_noprep$model$PID)),  summary(mod_def_noprep)$p.coef[["phase2"]],  summary(mod_def_noprep)$se[["phase2"]],  summary(mod_def_noprep)$p.pv[["phase2"]],

    "Drop Blip", "Intact",       paste0("0-", TAU, " Weeks"),  length(unique(mod_int_noblip$model$PID)),  summary(mod_int_noblip)$p.coef[["phase1"]],  summary(mod_int_noblip)$se[["phase1"]],  summary(mod_int_noblip)$p.pv[["phase1"]],
    "Drop Blip", "Defective",    paste0("0-", TAU, " Weeks"),  length(unique(mod_def_noblip$model$PID)),  summary(mod_def_noblip)$p.coef[["phase1"]],  summary(mod_def_noblip)$se[["phase1"]],  summary(mod_def_noblip)$p.pv[["phase1"]],
    "Drop Blip", "Intact",       paste0(TAU, "-24 Weeks"),     length(unique(mod_int_noblip$model$PID)),  summary(mod_int_noblip)$p.coef[["phase2"]],  summary(mod_int_noblip)$se[["phase2"]],  summary(mod_int_noblip)$p.pv[["phase2"]],
    "Drop Blip", "Defective",    paste0(TAU, "-24 Weeks"),     length(unique(mod_def_noblip$model$PID)),  summary(mod_def_noblip)$p.coef[["phase2"]],  summary(mod_def_noblip)$se[["phase2"]],  summary(mod_def_noblip)$p.pv[["phase2"]],

    "Drop Inc.", "Intact",       paste0("0-", TAU, " Weeks"),  length(unique(mod_int_inc$model$PID)),     summary(mod_int_inc)$p.coef[["phase1"]],     summary(mod_int_inc)$se[["phase1"]],     summary(mod_int_inc)$p.pv[["phase1"]],
    "Drop Inc.", "Defective",    paste0("0-", TAU, " Weeks"),  length(unique(mod_def_inc$model$PID)),     summary(mod_def_inc)$p.coef[["phase1"]],     summary(mod_def_inc)$se[["phase1"]],     summary(mod_def_inc)$p.pv[["phase1"]],
    "Drop Inc.", "Intact",       paste0(TAU, "-24 Weeks"),     length(unique(mod_int_inc$model$PID)),     summary(mod_int_inc)$p.coef[["phase2"]],     summary(mod_int_inc)$se[["phase2"]],     summary(mod_int_inc)$p.pv[["phase2"]],
    "Drop Inc.", "Defective",    paste0(TAU, "-24 Weeks"),     length(unique(mod_def_inc$model$PID)),     summary(mod_def_inc)$p.coef[["phase2"]],     summary(mod_def_inc)$se[["phase2"]],     summary(mod_def_inc)$p.pv[["phase2"]],
    ) %>%
    mutate(PercentDecayRatePerWeek = -100*(2^Estimate - 1)) %>% # negative because it's in terms of decay rather than percent change
    mutate(halflife_week = -1/Estimate) %>%
    mutate(halflifesd = 1/Estimate^2*SE) %>%
    mutate(halflife_lowCI = halflife_week - 1.96*halflifesd, halflife_highCI = halflife_week + 1.96*halflifesd) %>%
    select(-halflifesd) %>% 
    kable()
```

| Model | Outcome | Phase | N\_participants | Estimate | SE | p\_value | PercentDecayRatePerWeek | halflife\_week | halflife\_lowCI | halflife\_highCI |
| --- | --- | --- | --- | --- | --- | --- | --- | --- | --- | --- |
| Week 0-24 | Intact | 0-5 Weeks | 61 | -0.3536168 | 0.0279818 | 0.0000000 | 21.7380349 | 2.827920 | 2.389323 | 3.266517 |
| Week 0-24 | Defective | 0-5 Weeks | 61 | -0.7351683 | 0.0515699 | 0.0000000 | 39.9252044 | 1.360233 | 1.173217 | 1.547249 |
| Week 0-24 | Intact | 5-24 Weeks | 61 | -0.0648236 | 0.0074178 | 0.0000000 | 4.3937772 | 15.426487 | 11.966585 | 18.886389 |
| Week 0-24 | Defective | 5-24 Weeks | 61 | 0.0065577 | 0.0137041 | 0.6326182 | -0.4555780 | -152.493087 | -777.100632 | 472.114457 |
| Week 0-52 | Intact | 0-5 Weeks | 61 | -0.3767948 | 0.0263945 | 0.0000000 | 22.9853298 | 2.653964 | 2.289579 | 3.018349 |
| Week 0-52 | Defective | 0-5 Weeks | 61 | -0.7373067 | 0.0480977 | 0.0000000 | 40.0141840 | 1.356288 | 1.182874 | 1.529702 |
| Week 0-52 | Intact | 5-24 Weeks | 61 | -0.0516874 | 0.0046970 | 0.0000000 | 3.5192799 | 19.347068 | 15.901150 | 22.792986 |
| Week 0-52 | Defective | 5-24 Weeks | 61 | 0.0134538 | 0.0085650 | 0.1171592 | -0.9369102 | -74.328282 | -167.073452 | 18.416888 |
| Drop PrEP | Intact | 0-5 Weeks | 37 | -0.4269394 | 0.0246924 | 0.0000000 | 25.6161875 | 2.342253 | 2.076739 | 2.607766 |
| Drop PrEP | Defective | 0-5 Weeks | 37 | -0.7815723 | 0.0610026 | 0.0000000 | 41.8267547 | 1.279472 | 1.083738 | 1.475206 |
| Drop PrEP | Intact | 5-24 Weeks | 37 | -0.0618360 | 0.0063871 | 0.0000000 | 4.1955889 | 16.171806 | 12.897827 | 19.445786 |
| Drop PrEP | Defective | 5-24 Weeks | 37 | 0.0008521 | 0.0158154 | 0.9570833 | -0.0590833 | -1173.515772 | -43862.333232 | 41515.301689 |
| Drop Blip | Intact | 0-5 Weeks | 57 | -0.3330279 | 0.0293368 | 0.0000000 | 20.6131416 | 3.002752 | 2.484300 | 3.521203 |
| Drop Blip | Defective | 0-5 Weeks | 57 | -0.7367017 | 0.0547310 | 0.0000000 | 39.9890239 | 1.357401 | 1.159747 | 1.555056 |
| Drop Blip | Intact | 5-24 Weeks | 57 | -0.0652396 | 0.0077279 | 0.0000000 | 4.4213436 | 15.328112 | 11.769400 | 18.886824 |
| Drop Blip | Defective | 5-24 Weeks | 57 | 0.0110600 | 0.0144691 | 0.4452608 | -0.7695701 | -90.415534 | -322.253370 | 141.422302 |
| Drop Inc. | Intact | 0-5 Weeks | 54 | -0.3709591 | 0.0224900 | 0.0000000 | 22.6731738 | 2.695715 | 2.375388 | 3.016043 |
| Drop Inc. | Defective | 0-5 Weeks | 54 | -0.7597576 | 0.0545105 | 0.0000000 | 40.9404432 | 1.316209 | 1.131118 | 1.501300 |
| Drop Inc. | Intact | 5-24 Weeks | 54 | -0.0648341 | 0.0060265 | 0.0000000 | 4.3944771 | 15.423974 | 12.613944 | 18.234004 |
| Drop Inc. | Defective | 5-24 Weeks | 54 | 0.0091502 | 0.0146429 | 0.5325567 | -0.6362599 | -109.287090 | -452.070359 | 233.496178 |

```
bind_rows(
    halflife(mod_int, 1) %>% 
        mutate(Outcome = "Intact", 
               Phase = paste0("0-", TAU, " Weeks"),
               N_participants = length(unique(mod_int$model$PID))),
    halflife(mod_def, 1) %>% 
        mutate(Outcome = "Defective", 
               Phase = paste0("0-", TAU, " Weeks"),
               N_participants = length(unique(mod_int$model$PID))),
    halflife(mod_int, 2) %>% 
        mutate(Outcome = "Intact", 
               Phase = paste0(TAU, "-24 Weeks"),
               N_participants = length(unique(mod_int$model$PID))),
    halflife(mod_def, 2) %>% 
        mutate(Outcome = "Defective", 
               Phase = paste0(TAU, "-24 Weeks"),
               N_participants = length(unique(mod_int$model$PID)))) %>%
    filter(grepl(pattern = "eid_art_weeks", x = term)) %>%
    mutate(estimate = estimate, std.error = std.error) %>%
    mutate(LB = estimate - 1.96*std.error, UB = estimate + 1.96*std.error) %>%
    mutate(p.value = 2*pnorm(abs(estimate/std.error), lower.tail = FALSE)) %>%
    select(Outcome, Phase, N_participants, `Increase in Half-life (Weeks) per Week Delay in ART Initiation` = estimate, LB, UB, p.value) %>%
    kable()
```

| Outcome | Phase | N\_participants | Increase in Half-life (Weeks) per Week Delay in ART Initiation | LB | UB | p.value |
| --- | --- | --- | --- | --- | --- | --- |
| Intact | 0-5 Weeks | 61 | 0.0826798 | 0.0202849 | 0.1450747 | 0.0093986 |
| Defective | 0-5 Weeks | 61 | 0.0579248 | 0.0312251 | 0.0846245 | 0.0000212 |
| Intact | 5-24 Weeks | 61 | 1.0775701 | 0.3160228 | 1.8391175 | 0.0055482 |
| Defective | 5-24 Weeks | 61 | 25.6888424 | -200.3701289 | 251.7478137 | 0.8237456 |

```
mean_vars = df %>% 
    select(PID, first_cd4, log10firstvl) %>%
    distinct() %>%
    select(-PID) %>%
    summarise(across(everything(), mean))

df %>% 
    select(PID, first_cd4, log10firstvl) %>%
    distinct() %>%
    pivot_longer(c(first_cd4, log10firstvl), names_to = "variable", values_to = "value") %>%
    summarise(
        p25 = quantile(value, probs = 0.25),
        p50 = quantile(value, probs = 0.50),
        p75 = quantile(value, probs = 0.75),
        .by = variable) %>%
    pivot_longer(cols = starts_with("p"), names_to = "quantile", values_to = "value") %>%
    mutate(var2 = variable) %>% 
    pivot_wider(id_cols = c(variable, quantile), names_from = var2, values_from = value) %>%
    mutate(first_cd4 = replace_na(first_cd4, mean_vars$first_cd4),
           log10firstvl = replace_na(log10firstvl, mean_vars$log10firstvl)) %>%
    mutate(phase1 = 0, phase2 = 0, eid_art_weeks = 0, PID = "a") %>% # PID is later ignored
    nest_by(variable) %>% 
    mutate(int = list(as_tibble(predict.gam(mod_int, newdata = data, se.fit = TRUE, terms = paste0("s(", variable,")")))), 
           def = list(as_tibble(predict.gam(mod_def, newdata = data, se.fit = TRUE, terms = paste0("s(", variable,")"))))) %>% 
    pivot_longer(c(int, def), names_to = "assay", values_to = "values") %>% 
    unnest(c(data, values)) %>%
    mutate(value = case_match(variable, "first_cd4" ~ first_cd4, "log10firstvl" ~ log10firstvl), .after = quantile) %>%
    mutate(LB = fit - 1.96*se.fit, 
           UB = fit + 1.96*se.fit, 
           p.value = 2*pnorm(abs(fit/se.fit), lower.tail = FALSE)) %>%
    mutate(across(fit:UB, ~.x*log2(10))) %>% # make log10 scale
    select(variable, assay, quantile, value, fit, se.fit, LB, UB, p.value) %>%
    arrange(variable, desc(assay), quantile) %>%
    kable()
```

| variable | assay | quantile | value | fit | se.fit | LB | UB | p.value |
| --- | --- | --- | --- | --- | --- | --- | --- | --- |
| first\_cd4 | int | p25 | 350.000000 | 1.89743862 | 0.80258286 | 0.32437621 | 3.4705010183 | 1.807074e-02 |
| first\_cd4 | int | p50 | 505.000000 | -0.04978915 | 0.02499204 | -0.09877355 | -0.0008047545 | 4.634907e-02 |
| first\_cd4 | int | p75 | 663.750000 | -2.04423474 | 0.86478252 | -3.73920848 | -0.3492610044 | 1.808508e-02 |
| first\_cd4 | def | p25 | 350.000000 | 1.26263170 | 0.54280567 | 0.19873258 | 2.3265308155 | 2.001209e-02 |
| first\_cd4 | def | p50 | 505.000000 | -0.14350055 | 0.25794593 | -0.64907456 | 0.3620734684 | 5.779919e-01 |
| first\_cd4 | def | p75 | 663.750000 | -1.44481147 | 0.62475430 | -2.66932990 | -0.2202930410 | 2.074423e-02 |
| log10firstvl | int | p25 | 3.783281 | -4.11577883 | 0.74805504 | -5.58196671 | -2.6495909571 | 3.755639e-08 |
| log10firstvl | int | p50 | 4.860373 | 0.09869849 | 0.02508246 | 0.04953687 | 0.1478601076 | 8.321035e-05 |
| log10firstvl | int | p75 | 5.669947 | 3.26634090 | 0.59353204 | 2.10301809 | 4.4296637085 | 3.729042e-08 |
| log10firstvl | def | p25 | 3.783281 | -3.71173449 | 0.87789060 | -5.43240007 | -1.9910689179 | 2.357622e-05 |
| log10firstvl | def | p50 | 4.860373 | -0.76218827 | 0.57050720 | -1.88038238 | 0.3560058406 | 1.815546e-01 |
| log10firstvl | def | p75 | 5.669947 | 2.14969912 | 0.57164524 | 1.02927445 | 3.2701237943 | 1.695418e-04 |

```
tribble(
    ~Model,      ~Outcome,       ~Phase,                       ~N_participants,                           ~Estimate,                                   ~SE,                                     ~p_value,
    "Week 0-24", "Plasma VL",       paste0("0-", TAU1, " Weeks"),  length(unique(mod_pvl$model$PID)),         summary(mod_pvl)$p.coef[["phase1"]],         summary(mod_pvl)$se[["phase1"]],         summary(mod_pvl)$p.pv[["phase1"]],
    "Week 0-24", "Plasma VL",    paste0(TAU1, "-", TAU2, " Weeks"),  length(unique(mod_pvl$model$PID)),         summary(mod_pvl)$p.coef[["phase2"]],         summary(mod_pvl)$se[["phase2"]],         summary(mod_pvl)$p.pv[["phase2"]],
    "Week 0-24", "Plasma VL",       paste0(TAU2, "-24 Weeks"),     length(unique(mod_pvl$model$PID)),         summary(mod_pvl)$p.coef[["phase3"]],         summary(mod_pvl)$se[["phase3"]],         summary(mod_pvl)$p.pv[["phase3"]],
    ) %>%
    mutate(PercentDecayRatePerDay = -100*(2^(Estimate/7) - 1)) %>% # negative because it's in terms of decay rather than percent change
    mutate(halflife_days = -1/Estimate*7) %>%
    mutate(halflifesd = 1/Estimate^2*SE*7) %>%
    mutate(halflife_lowCI = halflife_days - 1.96*halflifesd, halflife_highCI = halflife_days + 1.96*halflifesd) %>%
    select(-halflifesd) %>% 
    kable()
```

| Model | Outcome | Phase | N\_participants | Estimate | SE | p\_value | PercentDecayRatePerDay | halflife\_days | halflife\_lowCI | halflife\_highCI |
| --- | --- | --- | --- | --- | --- | --- | --- | --- | --- | --- |
| Week 0-24 | Plasma VL | 0-0.5 Weeks | 67 | -10.615739 | 0.9735147 | 0.0000000 | 65.047539 | 0.6593983 | 0.5408771 | 0.7779196 |
| Week 0-24 | Plasma VL | 0.5-4 Weeks | 67 | -1.418698 | 0.1399811 | 0.0000000 | 13.105976 | 4.9341020 | 3.9798924 | 5.8883115 |
| Week 0-24 | Plasma VL | 4-24 Weeks | 67 | -0.006499 | 0.0165690 | 0.6950879 | 0.064333 | 1077.0890923 | -4305.0853928 | 6459.2635773 |

```
bind_rows(
    halflife(mod_pvl, 1) %>% 
        mutate(Outcome = "Plasma VL", 
               Phase = paste0("0-", TAU1, " Weeks"),
               N_participants = length(unique(mod_pvl$model$PID))),
    halflife(mod_pvl, 2) %>% 
        mutate(Outcome = "Plasma VL", 
               Phase = paste0(TAU1, "-", TAU2, " Weeks"),
               N_participants = length(unique(mod_pvl$model$PID))),
    halflife(mod_pvl, 3) %>% 
        mutate(Outcome = "Plasma VL", 
               Phase = paste0(TAU2, "-24 Weeks"),
               N_participants = length(unique(mod_pvl$model$PID))),
    ) %>%
    filter(grepl(pattern = "eid_art_weeks", x = term)) %>%
    mutate(estimate = estimate, std.error = std.error) %>%
    mutate(LB = estimate - 1.96*std.error, UB = estimate + 1.96*std.error) %>%
    mutate(p.value = 2*pnorm(abs(estimate/std.error), lower.tail = FALSE)) %>%
    select(Outcome, Phase, N_participants, `Change in Half-life (Days) per Week Delay in ART Initiation` = estimate, LB, UB, p.value) %>%
    kable()
```

| Outcome | Phase | N\_participants | Change in Half-life (Days) per Week Delay in ART Initiation | LB | UB | p.value |
| --- | --- | --- | --- | --- | --- | --- |
| Plasma VL | 0-0.5 Weeks | 67 | -0.0049234 | -0.0074286 | -0.0024182 | 0.0001172 |
| Plasma VL | 0.5-4 Weeks | 67 | 0.0426776 | 0.0180335 | 0.0673216 | 0.0006882 |
| Plasma VL | 4-24 Weeks | 67 | 53.7437700 | -504.8544859 | 612.3420259 | 0.8504258 |

```
df %>% 
    select(PID, first_cd4) %>%
    distinct() %>%
    summarise(
        p25 = quantile(first_cd4, probs = 0.25),
        p50 = quantile(first_cd4, probs = 0.50),
        p75 = quantile(first_cd4, probs = 0.75)) %>%
    pivot_longer(cols = starts_with("p"), names_to = "quantile", values_to = "first_cd4") %>%
    mutate(phase1 = 0, phase2 = 0, phase3 = 0, eid_art_weeks = 0, PID = "a") %>%
    cbind(as_tibble(predict.gam(mod_pvl, newdata = ., se.fit = TRUE, terms = "s(first_cd4)"))) %>%
    mutate(variable = "Initial CD4 Count", .before = quantile) %>%
    mutate(LB = fit - 1.96*se.fit, 
           UB = fit + 1.96*se.fit, 
           p.value = 2*pnorm(abs(fit/se.fit), lower.tail = FALSE)) %>%
    mutate(across(fit:UB, ~.x*log2(10))) %>% # make log10 scale
    select(variable, quantile, first_cd4, fit, se.fit, LB, UB, p.value) %>%
    kable()
```

| variable | quantile | first\_cd4 | fit | se.fit | LB | UB | p.value |
| --- | --- | --- | --- | --- | --- | --- | --- |
| Initial CD4 Count | p25 | 350.00 | -0.4640119 | 0.8252458 | -2.0814936 | 1.153470 | 0.5739313 |
| Initial CD4 Count | p50 | 505.00 | 0.3013208 | 0.9641549 | -1.5884229 | 2.191064 | 0.7546429 |
| Initial CD4 Count | p75 | 663.75 | 1.0924697 | 0.9336179 | -0.7374214 | 2.922361 | 0.2419420 |

# Exploratory Data Analysis/Visualization

```
ggarrange(
    df %>%
        select(PID, weeks_art_num, eid_art_days, first_cd4, log10firstvl, Intact, Defective) %>%
        filter(eid_art_days + avg_eid_art_days < 100) %>%
        pivot_longer(c(Intact, Defective), names_to = "assay", values_to = "value") %>%
        mutate(dynamics = case_when(
            eid_art_days + avg_eid_art_days < 30 ~ "<30 days",
            eid_art_days + avg_eid_art_days >= 30 | eid_art_days + avg_eid_art_days < 100  ~ "30-100 days"
        )) %>%
        mutate(assay = factor(assay, levels = c("Intact", "Defective"), labels = c("HIV Intact DNA", "HIV Defective DNA"))) %>%
        arrange(PID, weeks_art_num) %>%
        ggplot(aes(x = weeks_art_num, y = value, linetype = dynamics)) + 
        facet_wrap(~assay) + 
        geom_path(aes(group = PID), col = "lightgray") +
        geom_smooth(method = "loess", col = "black", se = FALSE, formula = y ~ x) + 
        scale_x_continuous(name = "Sampling Timepoints (Weeks on ART)", breaks = c(2,(0:6)*4)) +
        scale_y_log10(name = expression(atop("HIV DNA", paste("(copies/", 10^6, " CD4+ T cells)")))) +
        labs(linetype = "Timing of ART Initiation") +
        theme_pubr(base_size = 5, base_family = "Helvetica"),
    df %>%
        select(PID, weeks_art_num, eid_art_days, first_cd4, log10firstvl, Intact, Defective) %>%
        pivot_longer(c(Intact, Defective), names_to = "assay", values_to = "value") %>%
        mutate(dynamics = cut_number(first_cd4, n = 3, dig.lab = 4)) %>%
        mutate(assay = factor(assay, levels = c("Intact", "Defective"), labels = c("HIV Intact DNA", "HIV Defective DNA"))) %>%
        arrange(PID, weeks_art_num) %>%
        ggplot(aes(x = weeks_art_num, y = value, linetype = dynamics)) + 
        facet_wrap(~assay) + 
        geom_path(aes(group = PID), col = "lightgray") +
        geom_smooth(method = "loess", col = "black", se = FALSE, formula = y ~ x) + 
        scale_x_continuous(name = "Sampling Timepoints (Weeks on ART)", breaks = c(2,(0:6)*4)) +
        scale_y_log10(name = expression(atop("HIV DNA", paste("(copies/", 10^6, " CD4+ T cells)")))) +
        labs(linetype = expression("Initial CD4+ T Cell Count (Cells/" ~ mm^3 * ")")) +
        theme_pubr(base_size = 5, base_family = "Helvetica"),
    df %>%
        select(PID, weeks_art_num, eid_art_days, first_cd4, log10firstvl, Intact, Defective) %>%
        pivot_longer(c(Intact, Defective), names_to = "assay", values_to = "value") %>%
        mutate(dynamics = cut_number(log10firstvl, n = 3)) %>%
        mutate(assay = factor(assay, levels = c("Intact", "Defective"), labels = c("HIV Intact DNA", "HIV Defective DNA"))) %>%
        arrange(PID, weeks_art_num) %>%
        ggplot(aes(x = weeks_art_num, y = value, linetype = dynamics)) + 
        facet_wrap(~assay) + 
        geom_path(aes(group = PID), col = "lightgray") +
        geom_smooth(method = "loess", col = "black", se = FALSE, formula = y ~ x) + 
        scale_x_continuous(name = "Sampling Timepoints (Weeks on ART)", breaks = c(2,(0:6)*4)) +
        scale_y_log10(name = expression(atop("HIV DNA", paste("(copies/", 10^6, " CD4+ T cells)")))) +
        labs(linetype = expression(Log[10] ~ "Pre-ART Viral Load (Cells/mL)")) +
        theme_pubr(base_size = 5, base_family = "Helvetica"),
    nrow = 3,
    labels = c("a.", "b.", "c.")
)
```

```
ggsave("figs/main/observed_ipda_decays.pdf", width = 88, height = 150, units = "mm")
```

```
ggarrange(
    df_full %>% 
        filter(weeks_art_num < 28) %>%
        bind_rows(tribble(
            ~PID,                                    ~gender, ~weeks_art_num, ~Intact, ~Defective,
            "c58445ff-f5a8-4a45-8c64-84b1875dfd14", "female",           5.14,    23.8,       35.6, # low viability sample
            "c58445ff-f5a8-4a45-8c64-84b1875dfd14", "female",             24,    23.5,       43.8, # linear interpolation of week 11 and week 36
        )) %>%
        pivot_longer(cols = c(Intact, Defective), names_to = "assay", values_to = "count") %>%
        mutate(assay = factor(assay, levels = c("Intact", "Defective"), labels = c("HIV Intact DNA", "HIV Defective DNA"))) %>%
        mutate(gender = factor(gender, levels = c("male", "female", "transfem"), labels = c("Male", "Cisgender Female", "Transgender Female"))) %>%
        ggplot(aes(x = weeks_art_num, y = count)) +
        facet_wrap(~assay, scales = "fixed") +
        geom_line(aes(group = PID, color = gender)) +
        labs(x = "Sampling Timepoints (Weeks on ART)", 
             y = expression(atop("HIV DNA", "(copies /" * 10^6 * " CD4+ T Cells)")), 
             color = "") +
        scale_x_continuous(breaks = c(2,(0:6)*4)) +
        scale_y_log10() +
        scale_color_manual(values = c("#99999950", "#E69F00", "#56B4E9")) + 
        theme_pubr(base_size = 11, base_family = "Helvetica", legend = "top"),
    df %>%
        pivot_longer(cols = c(Intact, Defective), names_to = "assay", values_to = "count") %>%
        mutate(assay = factor(assay, levels = c("Intact", "Defective"), labels = c("HIV Intact DNA", "HIV Defective DNA"))) %>%
        mutate(prep_history = as.factor(ifelse(prep_group == 2, 0, prep_group))) %>%
        ggplot(aes(x = weeks_art_num, y = count)) +
        facet_wrap(~assay, scales = "fixed") +
        geom_line(aes(group = PID, color = factor(prep_history))) +
        labs(x = "Sampling Timepoints (Weeks on ART)", 
             y = expression(atop("HIV DNA", "(copies /" * 10^6 * " CD4+ T Cells)")), 
             color = "") +
        scale_x_continuous(breaks = c(2,(0:6)*4)) +
        scale_y_log10() +
        scale_color_manual(
            values = c("#99999950", "#E69F00", "#56B4E9"),
            breaks = c(0, 1, 3),
            labels = c("No PrEP Overlap With\nHIV at Time of Diagnosis", 
                       "Aquired HIV While on PrEP", 
                       "PrEP Prescribed\nAfter HIV Aquisition")) + 
        theme_pubr(base_size = 11, base_family = "Helvetica", legend = "top"),
    nrow = 2,
    labels = c("a.", "b.")
)
```

```
ggsave("figs/supp/observed_decays_by_gender_and_prep.pdf", width = 6.5, height = 5, units = "in")
```

```
ggarrange(
    dfpvl %>%
        select(PID, weeks_art_num, eid_art_days, first_cd4, PlasmaVL) %>%
        filter(eid_art_days + avg_eid_art_days < 100) %>%
        mutate(dynamics = case_when(
            eid_art_days + avg_eid_art_days < 30 ~ "<30 days",
            eid_art_days + avg_eid_art_days >= 30 | eid_art_days + avg_eid_art_days < 100  ~ "30-100 days"
        )) %>%
        arrange(PID, weeks_art_num) %>%
        ggplot(aes(x = weeks_art_num, y = PlasmaVL, linetype = dynamics)) + 
        geom_path(aes(group = PID), col = "lightgray") +
        geom_smooth(method = "loess", col = "black", se = FALSE, formula = y ~ x) + 
        scale_y_log10() +
        scale_x_continuous(breaks = c(2,(0:6)*4)) +
        xlab("Sampling Timepoints (Weeks on ART)") +
        ylab("Plasma HIV RNA (copies/mL)") +
        labs(linetype = "Timing of ART Initiation") +
        theme_pubr(base_size = 11, base_family = "Helvetica"),
    dfpvl %>%
        select(PID, weeks_art_num, eid_art_days, first_cd4, PlasmaVL) %>%
        mutate(dynamics = cut_number(first_cd4, n = 3, dig.lab = 4)) %>%
        arrange(PID, weeks_art_num) %>%
        ggplot(aes(x = weeks_art_num, y = PlasmaVL, linetype = dynamics)) + 
        geom_path(aes(group = PID), col = "lightgray") +
        geom_smooth(method = "loess", col = "black", se = FALSE, formula = y ~ x) + 
        scale_y_log10() +
        scale_x_continuous(breaks = c(2,(0:6)*4)) +
        xlab("Sampling Timepoints (Weeks on ART)") +
        ylab("Plasma HIV RNA (copies/mL)") +
        labs(linetype = expression("Initial CD4+ T Cell Count (Cells/" * mm^3 * ")")) +
        theme_pubr(base_size = 11, base_family = "Helvetica"),
    nrow = 2,
    labels = c("a.", "b.")
)
```

```
ggsave("figs/supp/observed_decays_plasmavl.pdf", width = 6.5, height = 6, units = "in")
```

```
ggarrange(
    read_csv("anonymized_merged.csv") %>%
        mutate(fiebig_keele = factor(fiebig_keele, levels = c("I", "II", "III", "IV", "V"))) %>%
        distinct(PID, fiebig_keele) %>%
        summarise(perc = round(n()/67*100,1), .by = fiebig_keele) %>%
        ggplot(aes(x = "", y = perc, fill = fiebig_keele, label = paste0(perc, "%"))) +
        geom_col(color = "black") + 
        geom_text(position = position_stack(vjust = 0.5), size = 2) +
        coord_polar(theta = "y") +
        scale_x_discrete(name = NULL, breaks = NULL) +
        scale_y_continuous(name = NULL, breaks = NULL) +
        scale_fill_discrete(name = "Fiebig Stage") +
        theme_pubclean(base_size = 6, base_family = "Helvetica") +
        theme(legend.position = "right"),
    read_csv("anonymized_merged.csv") %>%
        mutate(ethnicity = factor(ethnicity, levels = c("WH", "ZZ", "LA", "AS", "AA"), labels = c("White", "Other", "Latinx", "Asian", "Black"))) %>%
        distinct(PID, ethnicity) %>%
        summarise(perc = round(n()/67*100,1), .by = ethnicity) %>%
        ggplot(aes(x = "", y = perc, fill = ethnicity, label = paste0(perc, "%"))) +
        geom_col(color = "black") + 
        geom_text(position = position_stack(vjust = 0.5), size = 2) +
        coord_polar(theta = "y") +
        scale_x_discrete(name = NULL, breaks = NULL) +
        scale_y_continuous(name = NULL, breaks = NULL) +
        scale_fill_discrete(name = "Ethnicity") +
        theme_pubclean(base_size = 6, base_family = "Helvetica") +
        theme(legend.position = "right"),
    ncol = 1,
    labels = c("a.", "b.")
)
```

```
ggsave("figs/main/demographics.pdf", width = 88, height = 120, units = "mm")
```
