# Supplementary material for: Rapid biphasic decay of intact and defective HIV DNA reservoir during acute treated HIV disease
Source: Nat Commun. 2024 Nov 18;15:9966. doi: 10.1038/s41467-024-54116-1 (PMC11574060; doi:10.1038/s41467-024-54116-1)
Supplement: Supplementary file 4 — Reporting Summary [file 41467_2024_54116_MOESM4_ESM.pdf]

nature portfolio

Corresponding author(s): Sulggi Lee and Alton Barbehenn

Last updated by author(s): Oct 3, 2024

Reporting Summary

Nature Portfolio wishes to improve the reproducibility of the work that we publish. This form provides structure for consistency and transparency in reporting. For further information on Nature Portfolio policies, see our Editorial Policies and the Editorial Policy Checklist.

Statistics

For all statistical analyses, confirm that the following items are present in the figure legend, table legend, main text, or Methods section.

n/a	Confirmed	
☐	☒	The exact sample size (*n*) for each experimental group/condition, given as a discrete number and unit of measurement
☐	☒	A statement on whether measurements were taken from distinct samples or whether the same sample was measured repeatedly
☐	☒	The statistical test(s) used AND whether they are one- or two-sided<br>*Only common tests should be described solely by name; describe more complex techniques in the Methods section.*
☐	☒	A description of all covariates tested
☐	☒	A description of any assumptions or corrections, such as tests of normality and adjustment for multiple comparisons
☐	☒	A full description of the statistical parameters including central tendency (e.g. means) or other basic estimates (e.g. regression coefficient) AND variation (e.g. standard deviation) or associated estimates of uncertainty (e.g. confidence intervals)
☐	☒	For null hypothesis testing, the test statistic (e.g. *F*, *t*, *r*) with confidence intervals, effect sizes, degrees of freedom and *P* value noted<br>*Give P values as exact values whenever suitable.*
☒	☐	For Bayesian analysis, information on the choice of priors and Markov chain Monte Carlo settings
☒	☐	For hierarchical and complex designs, identification of the appropriate level for tests and full reporting of outcomes
☒	☐	Estimates of effect sizes (e.g. Cohen's *d*, Pearson's *r*), indicating how they were calculated

Our web collection on statistics for biologists contains articles on many of the points above.

Software and code

Policy information about availability of computer code

Data collection	No software was used for data collection.
Data analysis	R version 4.3.1 was used for all analyses. Model fitting model fitting was conducted with the R packages mgcViz_0.1.11 and lspline_1.0-0. Data manipulation was conducted with the R packages readr_2.1.4, tidyr_1.3.0, dplyr_1.1.4. Data visualization was conducted with the R packages ggplot2_3.4.4, mgcViz_0.1.11, patchwork_1.1.3, and ggpubr_0.6.0.

For manuscripts utilizing custom algorithms or software that are central to the research but not yet described in published literature, software must be made available to editors and reviewers. We strongly encourage code deposition in a community repository (e.g. GitHub). See the Nature Portfolio guidelines for submitting code & software for further information.

Data

Policy information about availability of data

All manuscripts must include a data availability statement. This statement should provide the following information, where applicable:

- Accession codes, unique identifiers, or web links for publicly available datasets
- A description of any restrictions on data availability
- For clinical datasets or third party data, please ensure that the statement adheres to our policy

The raw clinical data are protected and are not available due to data privacy laws. De-identified processed virologic and clinical data generated in this study are available and have been deposited in the Dryad database (doi:10.5061/dryad.q573n5tsd).

Code to reproduce our analyses and figures is provided in an R markdown file available through the Dryad and Zenodo databases (doi:10.5061/dryad.q573n5tsd).

Research involving human participants, their data, or biological material

Policy information about studies with human participants or human data. See also policy information about sex, gender (identity/presentation), and sexual orientation and race, ethnicity and racism.

Reporting on sex and gender	The research performed involved self-reported gender, and no exclusions were made to study enrollment based on gender. Consistent with our San Francisco-based HIV population, our study included 98% self-reported male participants as shown in Table 1. Nonetheless, every effort was made to recruit and study females in this study. Sensitivity analyses were performed with and without self-reported non-male participants and since results were unchanged, data for the publication are presented for the total population as a whole. Source data are provided, including individual-level data included these data.
Reporting on race, ethnicity, or other socially relevant groupings	The research performed involved self-reported race/ethnicity, and no exclusions were made to study enrollment based on race/ethnicity. Consistent with national acute HIV incidence trends, our study consisted of a large proportion of non-White study participants: 15% Black; 30% Latino; 21% Asian; 32% White as shown in Table 1 and Fig. 1. Sensitivity analyses were performed by race/ethnicity groups, as well as by using race/ethnicity as a covariate in statistical models, but combined data are presented given that the overall findings were unchanged. Individual-level source data are included on our data on Dryad.
Population characteristics	The UCSF Treat Acute HIV study included adult participants, age 18 years and older, enrolled from December 1, 2015 to November 30, 2020 in the San Francisco Bay Area. A total of 67 (83% of those screened) were eligible for study. The proportions of Fiebig I, II, III, IV, V acute HIV staging were 12%, 15%, 8%, 17%, and 48%. The virologic suppression rate was 96% among N=57 participants retained in study by week 24; their current median follow-up is 1.3 (IQR:0.5-2.7) years.
Recruitment	Participants were recruited leveraging the San Francisco Department of Public Health's "Getting to Zero" campaign, aimed at identifying and providing immediate ART to newly diagnosed HIV+ individuals. Individuals with newly diagnosed acute (<100 days of) HIV infection were rapidly identified and eligible individuals were referred to the study. Participants were consented, provided immediate ART (tenofovir/emtricitabine + dolutegravir), and linked to clinical care. There were no differences in retention rates by unstable housing, mental health illness, or substance use disorder.
Ethics oversight	All participants provided written informed consent, and the institutional review board of the University of California San Francisco approved the research.

Note that full information on the approval of the study protocol must also be provided in the manuscript.

Field-specific reporting

Please select the one below that is the best fit for your research. If you are not sure, read the appropriate sections before making your selection.

- [x] Life sciences
- [ ] Behavioural & social sciences
- [ ] Ecological, evolutionary & environmental sciences

For a reference copy of the document with all sections, see nature.com/documents/nr-reporting-summary-flat.pdf

Life sciences study design

All studies must disclose on these points even when the disclosure is negative.

Sample size	Given our success in enrolling the first 24 participants in less than 12 months, we then continued to enroll an additional 36 participants over a 24 month period. Of the first 24 participants enrolled, 9 (38%) initiated ART ≤30 days from EDDI while 18 (75%) initiated ART ≤90 days of the estimated date of detected infection (EDDI). Based on prior data of early treated people with HIV from our San Francisco-based study population, we estimated that if assuming equally sized groups (i.e., N=30 participants treated during ≤30 vs. N=30 participants treated >30 days from EDDI), and a Type I error rate of 5%, that we would have greater than 80% power to detect a 45.5% reduction in cell-associated total HIV-1 DNA from PBMCs in participants treated within the first 30 days of HIV infection. The current analysis used the Intact Proviral DNA Assay (IPDA) from peripheral CD4+ T cells which includes much more accurate estimation of intact, defective, and total HIV DNA from the cellular subtype that harbors the majority of HIV. For these reasons, these earlier power calculations likely underestimated our ability to detect significant associations.
Data exclusions	Data were excluded from the primary analysis if the IPDA assay failed to produce confident measurements (we used a threshold of 0.7 for CD4+ T Cell Viability). This exclusion criteria were pre-established based on prior work with IPDA data.
Replication	Sensitivity analyses were performed for various clinical subcategories (e.g., by prior pre-exposure prophylaxis [PrEP] or post-exposure prophylaxis [PEP] use and viral control) and using samples up to a year on ART. Our main findings were replicated in the sensitivity analyses.
Randomization	NA – this was not a randomized clinical trial.
Blinding	NA – this was not a randomized clinical trial.

Reporting for specific materials, systems and methods

We require information from authors about some types of materials, experimental systems and methods used in many studies. Here, indicate whether each material, system or method listed is relevant to your study. If you are not sure if a list item applies to your research, read the appropriate section before selecting a response.

Materials & experimental systems

n/a	Involved in the study
☒	☐ Antibodies
☒	☐ Eukaryotic cell lines
☒	☐ Palaeontology and archaeology
☒	☐ Animals and other organisms
☒	☐ Clinical data
☒	☐ Dual use research of concern
☒	☐ Plants

Methods

n/a	Involved in the study
☒	☐ ChIP-seq
☒	☐ Flow cytometry
☒	☐ MRI-based neuroimaging

Plants

Seed stocks	*Report on the source of all seed stocks or other plant material used. If applicable, state the seed stock centre and catalogue number. If plant specimens were collected from the field, describe the collection location, date and sampling procedures.*
Novel plant genotypes	*Describe the methods by which all novel plant genotypes were produced. This includes those generated by transgenic approaches, gene editing, chemical/radiation-based mutagenesis and hybridization. For transgenic lines, describe the transformation method, the number of independent lines analyzed and the generation upon which experiments were performed. For gene-edited lines, describe the editor used, the endogenous sequence targeted for editing, the targeting guide RNA sequence (if applicable) and how the editor was applied.*
Authentication	*Describe any authentication procedures for each seed stock used or novel genotype generated. Describe any experiments used to assess the effect of a mutation and, where applicable, how potential secondary effects (e.g. second site T-DNA insertions, mosiacism, off-target gene editing) were examined.*
